# Sexual selection purges mutation load, but not overall genetic diversity, decreasing vulnerability to extinction

**DOI:** 10.1073/pnas.2608675123

**Published:** 2026-09-08

**Authors:** Michael D. Pointer, Will J. Nash, Matthew J. G. Gage, Tracey Chapman, Alexei A. Maklakov, David S. Richardson

**Affiliations:** ^a^ Department of Biological Sciences, Department of Biological Sciences, University of East Anglia, Norwich Research Park, Norwich NR4 7TJ, United Kingdom; ^b^ Earlham Institute, Norwich Research Park, Norwich NR4 7UZ, United Kingdom

**Keywords:** sexual selection, mutation load, experimental evolution, population viability

## Abstract

Theory suggests sexual selection will enhance population viability by purging deleterious alleles. However, direct genomic evidence for this fundamental idea is scarce and contradictory. We combined long-term experimental evolution with whole-genome resequencing to directly test how sexual selection affects mutation load, genomic divergence, and extinction risk in small populations (maximum N_e_ = 40) of *Tribolium castaneum*. After 156 generations, populations evolving under strong sexual selection carried substantially fewer deleterious alleles than populations under weak sexual selection, based on both individual-level estimates of missense and nonsense variants and population-level R_xy_ analyses, indicating more efficient purging of deleterious alleles. In contrast, nucleotide diversity and runs of homozygosity were similar across treatments, indicating that purging acted most strongly on deleterious variation, and that reduced mutation load in these small populations under strong sexual selection was not explained by demographic effects. Importantly, population-level mutation load estimates best explained extinction risk under inbreeding, directly linking sexual selection to purging and population viability. Genome scans of high and low sexual selection populations revealed peaks of divergence, which included genes involved in courtship, sex discrimination, and seminal fluid proteins. Our results provide direct genomic evidence that sexual selection can reduce mutation load without eroding standing genetic diversity and thus adaptive potential, while driving adaptive divergence in reproductive traits. This beneficial purging may help explain the widespread prevalence of sexual reproduction in nature despite inherent costs and have important ramifications as to how we manage populations of conservation concern.

## Significance

A long standing prediction is that sexual selection can improve population health by biasing reproduction away from individuals with high deleterious mutation load. However, direct genomic evidence is scarce, and the indirect data available are contradictory. Now, using whole-genome resequencing of experimentally evolved beetle populations, we show that populations evolving under stronger sexual selection carry fewer inferred deleterious mutations and, importantly, that the amount of these deleterious mutations present predicts population extinction risk. Our results provide direct genomic support for this long-debated evolutionary process that may help explain the widespread prevalence of sexual reproduction in nature. Our findings also highlight the importance of allowing sexual selection to act in populations of conservation concern.

Sexual selection may be a key mechanism for purging deleterious variation from the genome (1–3), however, this remains contentious (4–6). If, as recently shown in black grouse, *Lyrurus tetrix* (7), the mating success of individuals is negatively correlated with the burden of deleterious variants they carry [mutation load; (8, 9)], sexual selection will amplify purifying selection via “genic capture” (10, 11). Theory predicts this process should reduce mutation load in sexual populations relative to asexual ones, and thereby lower extinction risk, particularly under inbreeding when recessive load is expressed (1, 12, 13). Several studies have used experimental approaches to address this question with mixed results (3, 14–21), but none directly test the effect of sexual selection on functionally characterized variation. Furthermore, recent work suggests that sexual selection does not affect overall mutation load, thus questioning the role of sexual selection in purging of deleterious alleles (21). Here, we provide a direct test of this theory by quantifying putatively deleterious and loss-of-function variants in small populations evolving with or without sexual selection and explicitly linking this proxy of genomic load to population extinction risk.

Experimental evolution studies have reported evidence that sexual selection appears to slow the accumulation of deleterious mutations or improve nonsexual fitness components (3, 14, 16–18, 20, 22), or, alternatively, that it yields little net fitness benefit and can exacerbate sexual antagonism (4, 5, 19, 21, 23). However, most of these studies infer mutation load indirectly from changes in phenotype or genome-wide diversity (but see refs. 3 and 21) and have not identified or functionally annotated variants underlying this load. Indirect evidence consistent with the purging of load under sexual selection also comes from phenotypic studies in *Tribolium castaneum* flour beetles. These studies inferred a reduction in load because populations evolved under elevated sexual selection showed evidence of increased performance across fitness-related traits (18, 24–27). For example, beetle lines under strong (vs. weak) sexual selection showed increased resistance to extinction under inbreeding (18). No study has yet directly quantified mutation load in experimental lines evolved under differing levels of sexual selection and then tested how that is linked to population viability, which is the major gap we address here.

Using whole-genome resequencing data from 84 individuals, we functionally annotated segregating variants and used the burden of putatively deleterious sites as a proxy of mutation load. These individuals came from the six *T. castaneum* lines described in Lumley et al. (18) that had been experimentally evolved in small populations for 156 generations under either monandrous (absent/weak sexual selection) or polyandrous (strong sexual selection) mating regimes. We directly determined genome-wide nucleotide diversity and estimated mutation load by categorizing mutations into functional categories (missense tolerated, missense deleterious, nonsense). We also determined relative mutation load at the population-level by comparison of allele frequencies across these different functional categories of variants. Alongside this, we identified loci where allele frequency differences were significantly associated with sexual selection regime, providing putative targets of selection across the genome. Using these loci to define highly differentiated regions of the genome, we compared signatures consistent with the functional impacts of purifying selection, identified in our earlier analyses, and those associated with the action of directional selection. In addition, using data on survival under inbreeding from the same lines (18) we tested for associations between mutation load and extinction probability. Importantly, this allowed us to test whether purging of load in high sexual selection lines is associated with their observed inbreeding resilience (18, 27).

Our results showed a striking elevation in the purging of genome-wide deleterious variation in response to sexual selection. This was not associated with any reduction in overall genetic diversity and so cannot be explained by demographic differences, such as differential inbreeding among the sexual selection regimes during experimental evolution. Instead, elevated sexual selection has resulted in the elimination of deleterious genetic variation. Importantly, the overall genome-wide load of such deleterious variants that remains after selection then best predicts population extinction risk in this experiment. In addition, genomic windows which diverge between replicated lines were explained by positive selection for beneficial alleles with predicted roles in reproductive biology, courtship behavior, sex discrimination, and male seminal fluid protein function.

## Results

### Sexual Selection Is Associated with a Lower Burden of Deleterious Variants.

**Fig. 1.**
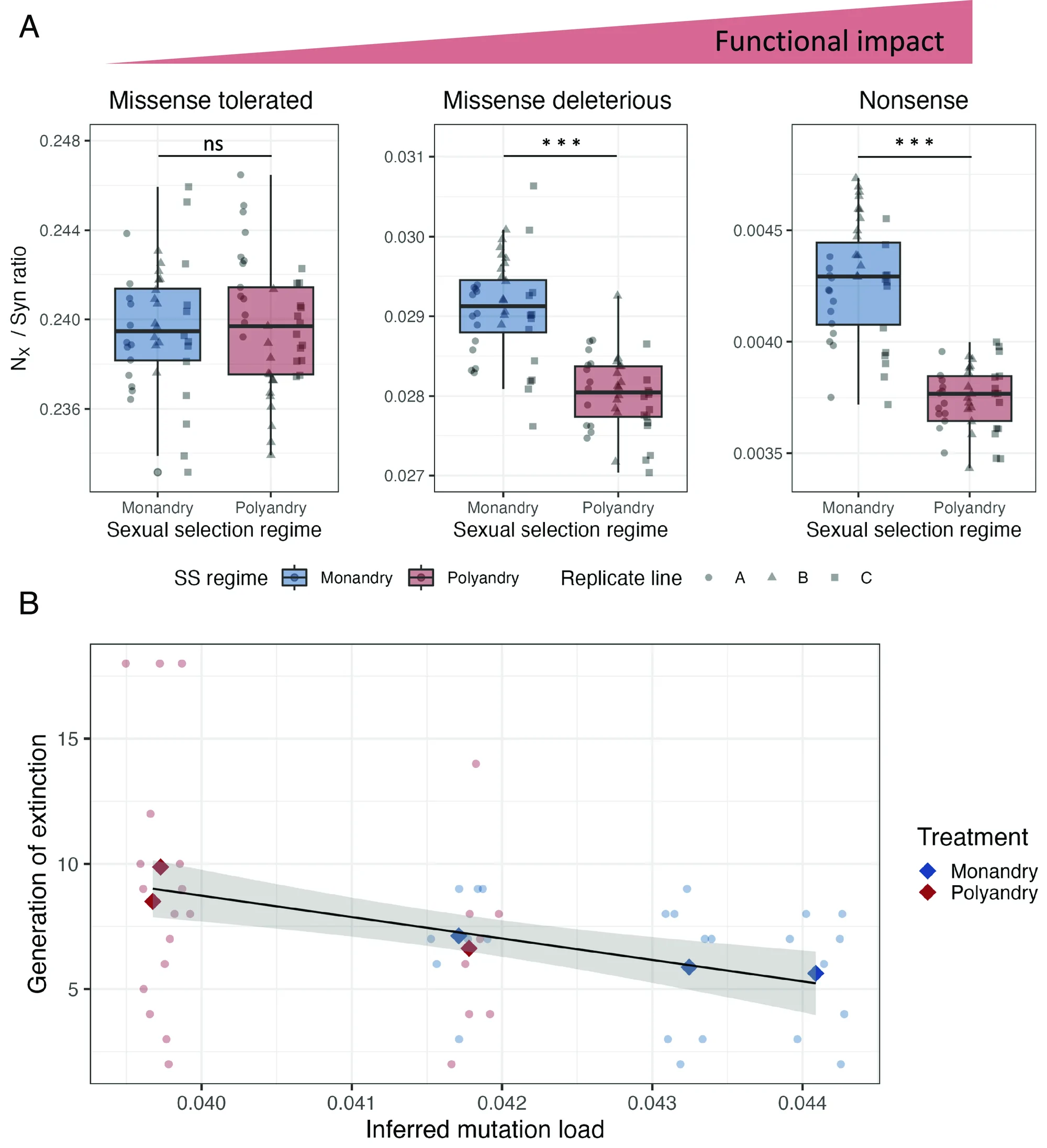
Strong sexual selection is associated with significantly reduced burden of putative deleterious variants in populations of *T. castaneum*, and this lower mutation load is associated with greater survival under inbreeding. (*A*) Genetic variation categorized by increasingly deleterious predicted functional impact (red wedge) in populations evolved under either monandrous or polyandrous sexual selection regimes for 156 generations. The ratio of missense tolerated, missense deleterious, and nonsense (N_X_) sites to synonymous sites, per individual. (*B*) Each small point represents an individual family line (each derived from a treatment population), plotted at the generation in which it went extinct against the mutation load estimated for its source population. Large diamonds show the mean extinction generation for each replicate population. Inferred mutation load was quantified as ML = 0.25(N × del_Het + 2^.^N × nonsense_Het) + N × del_Hom + 2^.^N × nonsense_Hom, where N× denotes the ratio of variants in a focal category to synonymous variants observed in the same population, accounting for partial recessivity of deleterious alleles and assigning nonsense variants twice the weight of missense deleterious variants (sensitivity analysis showed that the relationship was insensitive to these parameters—see *SI Appendix*). Points are colored by mating system treatment (monandry: blue; polyandry: red). The black line shows a linear regression fitted through population-level means, with the gray shaded band indicating the 95% CI. Note that mutation load was estimated at the population level; all family lines within a population therefore share the same x-value but are jittered to ensure they are visible.

We estimated mutation load in lines of *T. castaneum* beetles drawn from populations evolved under experimental regimes of strong or weak sexual selection for 156 generations (18). Briefly, these regimes either allowed polyandry, 5 males to 1 female, elevating the potential for SS; or enforced monandry, 1 male to 1 female, minimizing/removing the potential for SS while controlling for population size (*Materials and Methods*). Individual mutation load was estimated by quantifying variants in three functional categories: missense tolerated, missense deleterious, and nonsense. Variant counts were normalized by the number of synonymous variants in the same individual to obtain a ratio, accounting for variation in genetic diversity and sequencing coverage. These normalized per-individual counts of high-quality SNPs provide a relative measure of functional mutational burden, which we use as an estimate of mutation load in population-level comparisons. Considering the coding regions of autosomes, the ratio of missense tolerated variants relative to synonymous variants, per individual, was not significantly different between monandrous and polyandrous populations (β = −1.65 × 10^−5^, SE = 6.06 × 10^−4^, *P* = 0.98; Fig. 1*A*). For missense deleterious variants, ratios were significantly lower in polyandrous populations than in monandrous ones (β = −1.08 × 10^−3^, SE = 1.15 × 10^−4^, *P* < 0.0001). Populations evolved under polyandry carried fewer nonsense variants than those evolved under monandry (β = −5.23 × 10^−4^, SE = 3.98 × 10^−5^, *P* < 0.0001). A complementary population-level analysis based on R_xy_ yielded qualitatively similar patterns, indicating a relative enrichment of deleterious variants in monandrous compared with polyandrous lines (*SI Appendix*, Fig. S5 and *Supplementary results*).

Across autosomal loci, we found no evidence that the effect of sexual selection regime differed between males and females for any of the three functional variant categories (interaction term: *P* > 0.53). After removing the interaction term, sex did not predict the relative number of autosomal missense tolerated, or missense deleterious variants (*P* > 0.18; *SI Appendix*, Fig. S2). In contrast, sex was associated with the number of nonsense variants, with females carrying fewer than males (β = −1.38e^−4^, SE = 3.63 × 10^−5^, *P* < 0.01). *T. castaneum* has an X/Y sex determination system with males the heterogametic sex (28). On the X chromosome, the ratio of missense tolerated or missense deleterious variants to synonymous variants did not vary between sexual selection treatments (*P* > 0.07; *SI Appendix*, Fig. S3). However, polyandrous populations exhibited marginally more nonsense variants than monandrous populations (β = 2.37 × 10^−4^, SE = 1.16 × 10^−4^, *P* = 0.044). As on the autosomes, there was no evidence for differing effect of sexual selection regime on males and females across the three variant categories tested using the X chromosome (all *P* > 0.21). With the interaction term removed, sex was significantly associated with the relative number of missense tolerated variants, with lower counts in males (β = −1.09 × 10^−2^, SE = 2.03 × 10^−3^, *P* < 0.0001; *SI Appendix*, Fig. S4). A marginally significant association with sex, following the same pattern, was also observed for missense deleterious variants (β = −8.09 × 10^−4^, SE = 4.01 × 10^−4^, *P* < 0.04) but there was no difference in the relative number of nonsense variants (*P* = 0.94).

### Survival of Sexual Selection Lines Following Inbreeding Is More Parsimoniously Explained by Mutation Load Than by Sexual Selection Regime.

Higher mutation load is predicted to increase the risk of population extinction under inbreeding. Lumley et al. (18) showed that polyandrous populations had lower extinction vulnerability than monandrous populations, under enforced inbreeding, using the same experimental lines we employ in this study. To explore the link between mutation load and extinction vulnerability, we extended the survival analysis framework of Lumley et al. (18) to include our line-level genomic estimate of mutation load. Replicating their findings, sexual selection regime significantly predicted time to extinction, with polyandrous populations exhibiting lower extinction risk (Fig. 1*B* and Table 1). However, AIC model comparison showed strongest support for a model including our genomic estimate of mutation load alone (AIC = 276; AIC weight = 0.66; Table 1), compared to a model including sexual selection regime only (AIC = 280). When both predictors were included, mutation load remained marginally significant (*P* = 0.046), whereas the effect of sexual selection regime was nonsignificant (*P* = 0.90; AIC = 278). This drop in significance is expected, given that we know sexual selection regime and mutation load to covary but is further evidence that the genomic estimate of mutation load provides explanatory power beyond that captured by sexual selection regime.

**Table 1.** Survival under inbreeding is better explained by mutation load than by sexual selection regime

Model	SS regime effect (coef, *P*)	Mutation load effect (coef, *P*)	LokLik	AIC	AIC weight
SS regime	−0.730, *P* = 0.026	–	−138.0	280.0	0.087
Mutation load	–	299.5, *P* = 0.003	−136.0	276.0	0.667
SS regime + mutation load	0.065, *P* = 0.900	314.8, *P* = 0.046	−136.0	278.0	0.245

Functional characterization of genomic variants was used to estimate mutation load in 84 genomes sequenced from *T. castaneum* lines evolved under strong (polyandry) or weak (monandry) sexual selection (SS) regimes for 156 generations. Three mixed models (GLMM) were fitted to population survival data, when families derived from these same lines were subjected to enforced inbreeding [from Lumley et al. (18)], modeling survival as a product of mutation load or SS regime or both. Mutation load alone better explained survival than did SS regime, or a combined model.

### Sexual Selection does not Affect Overall Genome-Wide Variation.

We found no evidence that genome wide nucleotide diversity (π) differed between monandrous (0.004 ± 0.001) and polyandrous (0.004 ± 8.60 × 10^−4^) mating regimes (linear model, F^1,4^ = 0.022 *P* = 0.969). Nor did π in coding regions differ between monandrous (0.002 ± 8.52 × 10^−4^) and polyandrous (0.002 ± 5.19 × 10^−4^) mating regimes (linear model, F^1,4^ = <0.001 *P* = 0.993).

**Fig. 2.**
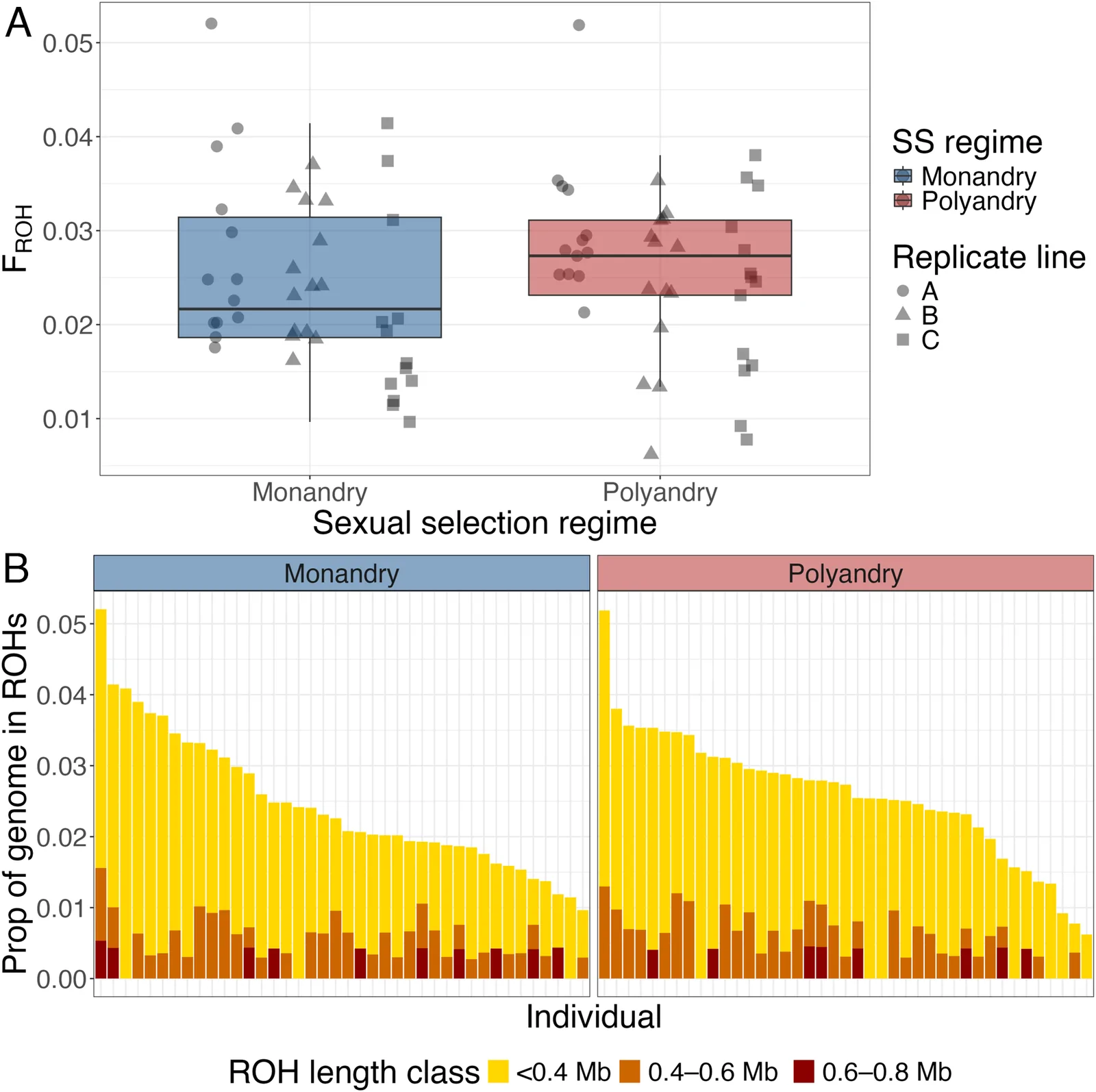
Sexual selection does not affect individual inbreeding. Inbreeding assessed via runs of homozygosity (ROH) in the genomes of *T. castaneum* experimentally evolved under monandrous and polyandrous sexual selection (SS) regimes. (*A*) genomic inbreeding coefficients, proportion of the genome present in ROH > 0.2 Mb. No significant difference between monandrous and polyandrous populations (LMM: β = 1.48 × 10^−3^, SE = 3.13 × 10^−3^, *P* = 0.662). (*B*) The proportion of the genome present in ROH of different lengths in each individual.

The genomic inbreeding coefficient (F_ROH_), computed, per individual, as the proportion of the callable genome in runs of homozygosity (>0.2 Mb), did not differ between monandrous and polyandrous regimes (0.024 ± 0.002 vs. 0.026 ± 0.001 respectively, β = 1.48 × 10^−3^, SE = 3.13 × 10^−3^, *P* = 0.662; Fig. 2*A*). The distribution of runs of homozygosity of different lengths also appeared similar between sexual selection regimes (Fig. 2*B*).

### Peaks of Genetic Divergence Between Populations Evolved Under High or Low Sexual Selection.

**Fig. 3.**
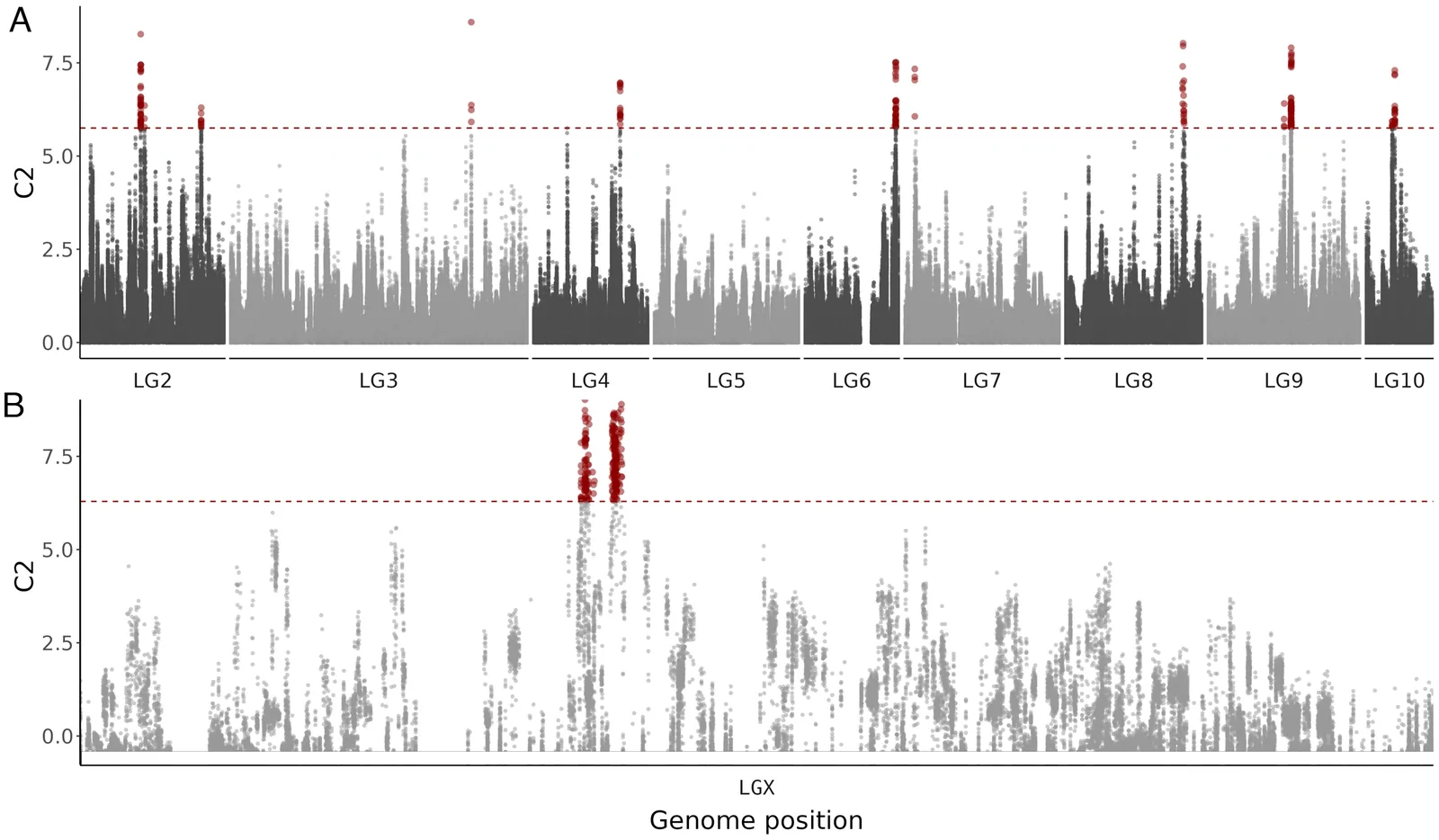
Genomic loci showing allele frequency differentiation associated with sexual selection regime in experimentally evolving *T. castaneum* populations. Values of the C2 statistic generated by BayPass analysis (29) measuring SNP-wise associations with the two sexual selection regimes (polyandrous vs. monandrous) while accounting for shared population history. Red points are in excess of the 0.999 quantile of C2 values from a BayPass run on a pseudo-observed dataset of putatively neutral SNPs, shown by the dotted line. Separate analyses were performed for (*A*) autosomal linkage groups, and (*B*) the X chromosome (LGX).

To identify genomic regions that had significantly diverged following 156 generations of experimental evolution under the two sexual selection regimes we performed a genome-wide scan for SNP-wise allele frequency differentiation, using BayPass (29). We identified 216 autosomal and 226 X chromosome SNPs as C2 outliers, grouped into 15 divergent windows genome-wide (Fig. 3 and *SI Appendix*, Table S3). Outlier SNPs on the X chromosome clustered into two strong peaks, separated by ~200 kb (Fig. 3*B*). Returning the closest gene to each of the 442 total candidate SNPs gave a set of 54 genes (*SI Appendix*, Table S3). Considering only genes located within 10 kb of just the top C2 SNP from each of the 15 candidate regions, 39 genes were identified (*SI Appendix*, Table S4), with 26 of these having well characterized homologs in *Drosophila melanogaster*.

Although neither gene set returned was enriched for gene ontology terms [G:profiler; (30)], background: all Tcas5.2 genes; *P* > 0.05), the 26 genes with functional information (from either the Tcas5.2 annotation or *D. melanogaster* homologues) reflected a diverse range of functions (*SI Appendix*, Table S4) including protein synthesis, lipid metabolism, and oxidative stress tolerance. 10 genes also had known functions (from the Tribolium 5.2 annotation or from Drosophila homologues) directly, or plausibly, related to reproductive biology, such as courtship behavior, sex discrimination, accessory gland proteins, and memory formation.

### Association Between Highly Divergent Regions and Deleterious Variants.

To test whether highly divergent genomic regions between populations under different selection regimes could be explained by the purging of deleterious variants, we characterized the SNPs within BayPass C2 outlier regions. Of 442 C2 outlier SNPs, 90 fell within coding sequences: 81 were synonymous, one was a nonsense variant on the X chromosome, and eight were missense, of which two were confidently predicted to be deleterious by SIFT (*SI Appendix*, Fig. S6).

We then tested whether these regions were enriched for deleterious variants that were purged under polyandry. Across 10,000 permutations, the observed number of purged deleterious variants overlapping outlier regions (2 variants) was lower than expected under the null distribution generated from synonymous variants (mean = 6.16, SD = 2.43). This deviation was not statistically significant (empirical *P* = 0.986, z = –1.71), indicating that highly divergent regions do not preferentially contain deleterious variants purged under polyandry. These results suggest that purifying selection against deleterious alleles is unlikely to be the primary driver of divergence in these regions.

## Discussion

Our results provide genomic evidence consistent with sexual selection enhancing the purging of deleterious alleles in small populations. Populations that evolved under strong sexual selection carried significantly fewer predicted deleterious variants, indicating reduced mutation load, than those where sexual selection was experimentally removed. This effect was consistent across both individual- and population-level analyses. Importantly, populations with a greater burden of putatively deleterious variants were more likely to go extinct when these populations were further inbred. These findings constitute the strongest and most comprehensive empirical support to date for theoretical predictions, and prior experimental observations, that sexual selection can facilitate the removal of deleterious mutations (1, 10).

The observed reduction in deleterious variants under polyandry indicates that sexual selection can act as a powerful source of purifying selection. Although our experimental design equalized the census-based effective population size between sexual selection regimes, we would have expected realized N_e_ to have been reduced under polyandry due to variance in male mating success. However, the absence of reduced π or increased F_ROH_ implies that any skew did not substantially alter genome-wide diversity. This pattern supports the idea that the observed reduction in inferred deleterious alleles in the sexual selection treatment was not just a by-product of demographic contraction or mating skew, but rather the outcome of more efficient selection, removing deleterious genetic variation per se. Under polyandry, both male–male competition and female choice likely increased the strength of selection against high-load males, reducing transmission of strongly deleterious variants without eroding neutral diversity [which at small N_e_ may also include low-medium deleterious variants (see below)]. The absence of N_e_ signatures therefore supports a model in which sexual selection enhances the efficacy of selection by favoring males carrying a lower burden of deleterious variation, rather than one in which purging arises from extreme reproductive skew.

The magnitude of purging scaled with the predicted functional impact of the variants assessed. Nonsense variants showed the strongest reduction under polyandry, followed by missense deleterious variants, while missense tolerated variants were largely unaffected. This pattern is consistent with the prediction that while sexual selection should help purge all deleterious alleles, it should be most effective against those with large fitness effects if mating success is sensitive to overall condition (1, 10). Our data provide genomic evidence to support this hypothesis. Nonsense mutations, which often lead to a complete loss-of-function, are likely highly deleterious and therefore more efficiently removed from the population when sexual selection is strong. Missense deleterious variants, which tend to have more moderate effects, were also reduced under polyandry, but to a lesser extent, and their frequency patterns approached those of missense tolerated variants. Essentially, sexual selection acts as a genome-wide filter that preferentially removes the most damaging coding variants, rather than uniformly reducing diversity.

Our study was performed using populations maintained at a small size (maximum N_e_ = 40) for many generations. Consequentially, due to inbreeding, low frequency recessive deleterious mutations hidden in heterozygote form in the ancestral outbred population are more likely to be exposed in homozygote form, hence providing more opportunity for purging. Indeed, evidence based on phenotypic assays does suggest that sexual selection helps purge inbreeding depression in small populations (15). The effects of sexual selection in large, outbred populations may be less efficient at purging mutation load (8). A second consequence of long-term low N_e_ would be higher levels of drift, such that less deleterious mutations would behave as if neutral. This would then help explain why purging scaled with the predicted functional impact of the variants assessed. Given that low–moderately deleterious mutations are probably a major component of standing genetic variation (31), this may explain why there is little effect of treatment on overall genetic diversity. However, these results must be treated with caution given that other experimental studies [undertaken with considerably larger population sizes (N = 1,000) than our study] have provided evidence that sexual selection does reduce diversity (3). Nevertheless, these processes may help explain the results we see in our experiment and suggest that such effects may be limited to smaller populations. This does not necessarily detract from the importance of these results, given that smaller/inbred populations are most prone to extinction, and thus where sexual selection may facilitate population persistence.

Despite sexual selection acting primarily through males in our experiment, we found that the females we sequenced carried fewer nonsense mutations than the males within the same treatment. This sex difference cannot be explained by overall purging of deleterious alleles, as both sexes share the same gene pool, but may instead reflect sex-specific selection pressures or mutational processes. For example, females with high mutational load may experience stronger viability selection prior to sampling meaning that only those with lower load would reach adulthood. Recent work has shown that deleterious mutations can have sex specific larval viability effects and that females are more sensitive than males to early life stress (32, 33). In humans, such effects may explain observed allele frequency differences between males and females, however it is important to note that these effects can only be relatively small (34, 35). Further work is required to assess the possibility of such mechanisms in our *T. castaneum* system. However, that mutation load of both sexes is reduced under polyandry does support the notion that sexual selection enhances purging primarily through male reproductive filtering, indirectly benefiting females and the population.

Our analysis of population persistence under inbreeding further supports the conclusion that sexual selection reduces mutation load in a way that enhances population viability, at least in small populations. This relationship parallels the earlier phenotypic findings of Lumley et al. (18), but our results now provide genomic evidence that populations evolving under strong sexual selection carry significantly fewer deleterious variants. Crucially, extinction risk was best predicted by our genomic estimates of mutation load, rather than sexual selection treatment itself, consistent with mutation load being the mechanistic link between sexual selection and extinction resilience. It is possible that a loss of balanced polymorphisms may also contribute to the effects we see under inbreeding. However, the consensus is that few variants segregating in a population are maintained by balancing selection, and that recessive deleterious mutations are the dominant cause of inbreeding depression (36). Remarkably, a proxy for mutation load derived from the experimental lines 102 generations after the inbreeding assay performed by Lumley et al. [(18); gens 54 vs. 156] still predicted past survival implying that differences in load between populations were both substantial and persistent. This temporal persistence may suggest that most purging took place early in the experimental evolution, prior to generation 54, but this is not possible to confirm with the samples/data available.

The findings from our study contribute to a growing recognition that sexual selection is important not only in evolutionary terms but also from a conservation perspective (22). By reducing mutation load while maintaining genomic diversity (but see ref. 3), sexual selection can enhance the resilience of small and/or inbred populations, potentially contributing to evolutionary rescue. Indeed, the purging of mutation load through sexual selection may contribute to resolving the sex paradox, explaining the long-term maintenance of sex in nature, despite its inherent costs (37). However, sexual selection also increases variance in reproductive success and so, in some situations, may exacerbate demographic stochasticity in small populations. Whether sexual selection ultimately helps or harms threatened populations will depend on the balance between its genetic benefits and demographic costs, which warrants future theoretical and empirical work.

In our study, genome scans revealed multiple peaks of divergence between populations evolved under differential strengths of sexual selection. The genes located in these divergent regions had diverse functions, including protein synthesis, lipid metabolism, and oxidative stress tolerance, as well as several that were directly linked to reproduction such as courtship behavior, sex discrimination, accessory gland proteins, and memory formation (38–40). These functions align with those seen to evolve in response to sexual selection in other systems (e.g., refs. 41–44). The diversity of functions suggests that experimental sexual selection influences a broad suite of physiological and behavioral traits, consistent with the polygenic nature of reproductive fitness, but focused on facets of reproductive biology key to both pre- and postmating reproductive success (recognition, courtship behavior as well as seminal fluid protein functions).

Importantly, divergent genomic regions were not enriched for deleterious variants that were at lower frequency in polyandrous populations. This suggests that genomic divergence between the polyandrous and monandrous populations is not the result of differential purging, but instead reflects positive selection for beneficial alleles, especially those associated with reproductive success. Our findings are consistent with the predictions of the “good genes” model, which proposes that sexual selection favors individuals carrying alleles that enhance fitness. Under the genic capture good genes model, sexually selected traits are condition dependent and reflect the cumulative effects of many loci (10, 11). In our *Tribolium* populations, polyandrous lines carried significantly fewer high-impact deleterious variants, particularly nonsense and deleterious missense alleles, indicating efficient genome-wide purging. At the same time, divergent genomic regions arising during experimental evolution were enriched for genes associated with reproductive functions but showed no enrichment for putatively deleterious variants, suggesting that adaptive divergence occurs largely independently of purging. Together, these results show that sexual selection can operate via two complementary processes: enhancing purifying selection on deleterious alleles across the genome while simultaneously driving adaptive differentiation at loci underpinning reproductive success. By linking functionally annotated mutation load to extinction risk in experimentally evolved populations, our results provide direct evidence of the genomic mechanisms by which sexual selection promotes both genomic health and adaptive divergence, thereby helping to maintain sexual reproduction and enhance population resilience.

In providing evidence that sexual selection reduces mutation load and increases population viability, our study supports the possibility that this mechanism may help explain the widespread prevalence of sexual reproduction in nature, despite the costs of such reproduction (37). The findings also highlight the importance of allowing sexual selection to act in sexually reproducing populations of conservation concern, given its potential to reduce mutation load without reducing overall genomic diversity.

## Materials and Methods

### Beetles and Husbandry.

Beetles were of the Georgia-1 (GA1) strain, collected from the wild in 1980 and kept by the Beeman laboratory at the US Department of Agriculture. Once acquired by our laboratory, the stock population was maintained on a standard medium of 90% organic wheat flour and 10% brewer’s yeast, which we refer to as “fodder,” at 30 °C, 60% relative humidity, and a 12:12 light–dark cycle (light from 0800-2000). The standard husbandry cycle consisted of two phases; during the oviposition phase, adult (12 ± 3 d posteclosion) beetles randomly chosen to parent the next generation were removed from their populations and placed into fresh fodder for 7 d of mating and egg-laying. Following this period, adults were sieved from the fodder and discarded, beginning a 35-d development phase—during which time, eggs in the fodder developed through larval and pupal stages to become adults. By preventing any interaction between sexually mature adults and offspring, this regime of nonoverlapping generations reduced the risk of negative density-dependent effects, eliminated opportunities for intergenerational interactions such as egg-cannibalism, and allowed accurate tracking of generations (45).

### Experimental Sexual Selection Regimes.

As described in Lumley et al. (18), unmated adult beetles from a single GA1 stock population (maintained at a size of 600 adults) were randomly assigned to begin each sexual selection regime. By manipulating the number of males and females housed together during the oviposition phase, differential levels of sexual selection were imposed on each regime while controlling for population size. The experiment consisted of six lines, representing three independent replicates to two selection regimes. Within each of three independent monandrous lines, the eggs produced by 20 independent single-pair matings were combined prior to the development phase. In three independent polyandrous lines, each of 12 females was housed with five males, with the eggs produced being combined for development. Thus, monandry removed all opportunity for female choice and male–male competition, whereas polyandry imposed sexual selection through competition between males for a single female, and provided that female with a choice between five males. The numbers of pairs used for monandry and groups used for polyandry were defined in order to equalize the effective population size, N_e_, for mixed adult sex ratios $\left(N_{e}=\frac{4N_{f}N_{m}}{N_{f}+N_{m}}\right)$ across treatments [(46); see also ref. 18]. Thus *N_e_* = 40 in both regimes. Treatments were applied for 156 beetle generations (15 y) prior to the commencement of this study.

### Sample Preparation and Sequencing.

Unmated adult beetles were collected from each replicate of the two sexual selection regimes, and flash-frozen in liquid nitrogen. For each individual DNA extraction was conducted using the DNeasy blood and tissue kit (insect tissue protocol, Qiagen), with the whole individual ground in liquid nitrogen. The extract was purified using a 1× bead cleanup with the AMPure XP SPRI protocol (Beckman Coulter). Library preparation and sequencing were performed at the Earlham Institute (Norwich, UK) using the low-input transposase-enabled (LITE) pipeline (*SI Appendix*, *Supplementary Methods*). Samples were sequenced on two flowcells across two sequencing runs, the first on the Illumina Novaseq 6000 and the second on the NovaSeq X Plus platform. Samples from all treatments were randomly distributed across both runs to avoid confounding treatment effects with sequencing platform or run-specific variation. Sequences were obtained from 84 individuals: 42 from each sexual selection regime, divided equally between each replicate line and between the sexes.

### Variant Calling and Filtering.

Reads were trimmed using Trimmomatic v0.39 (47) and mapped to the Tcas5.2 reference genome (48) using BWA-MEM v0.7.17 (49). Mapping was followed by SAMtools v1.18 fixmate and sort to ensure BAM files were correctly formatted and sorted prior to removing duplicates (50). PCR duplicates were removed using Picard v2.26.2 MarkDuplicates (51). Finally, mappings were filtered for complete read pairs with a mapping quality (MAPQ) >25 using SAMtools *view*. Joint genotyping was conducted using BCFtools v1.18.0 mpileup (50), with ploidy specified by sex and chromosome to ensure haploid genotyping of the X chromosome in males. BCFtools *call* was then used to call all sites under the multiallelic model (−m). BCFtools filter was used to remove variants within 3 bp of other variants, with a variant quality score <30, that were at a locus with sequencing depth less than 578 and greater than 5,201 (±3 times average sequencing depth), and were represented by data at that locus in less than 50% of individuals (−g 3 − G 3 −e′DP < 578||DP > 5,201||F_MISSING > 0.5||QUAL < 30′). The resulting file is referred to hereafter as the allsites vcf.

At this point, from the allsites vcf single nucleotide polymorphisms (SNPs) were extracted using BCFtools view and further filtered to remove sites with minor allele count <3. This file is referred to hereafter as the SNP vcf. To prepare the allsites vcf for further analysis, variant and invariant sites were handled separately: Invariant sites were extracted using VCFtools 0.16.0 (50) and stored in a separate file. Variant sites were filtered using VCFtools to only contain biallelic SNPs that did not deviate significantly from Hardy–Weinberg equilibrium (HWE *P*-value < 0.001). Following this filter, invariant and variant sites were concatenated using BCFtools *index* and *concat*. This file is referred to hereafter as the filtered allsites vcf.

Quality control of the SNP VCF revealed three samples (polyAmT1, monoCmT, monoAfT) with huge counts of singleton SNPs and indels compared to other samples. We excluded reads from these samples from the raw sequence data and reran the above steps to regenerate both the SNP vcf and the allsites VCF. After filtering, we took forward a set of 2,874,861 SNPs for analysis.

As samples were sequenced across two runs on different Illumina platforms, we used principal component analysis (PCA) to assess whether platform- or run-specific effects contributed to structure in the data. The SNP VCF was pruned for linkage with bcftools (+prune −m 0.3 −w 50 kb) before PCA with plink v1.9 (52) on a set of 62,713 SNPs. Using the first and second principal components generated by this analysis, which accounted for 29.1% and 19.6% of variance, respectively, we observed no clustering of samples by sequencing run (*SI Appendix*, Fig. S1); sequencing run was therefore not used as a covariate in subsequent analyses.

### Estimating Mutation Load.

SNPs within coding sequences (CDS) of the Tcas5.2 annotation were extracted and functionally annotated using SnpEff v4.3 (53), which assigned each variant to an effect category (nonsense, missense, or synonymous). Missense variants were further categorized using SIFT 4G (54), with the provided *T. castaneum* database, to predict the strength of their potential functional impact. SIFT uses a database of known proteins to score the putative effect of a change of amino acid at each position in the protein based on the frequency of that substitution in a multispecies alignment, and the biochemical similarity of the amino acids. Each SNP obtains a SIFT score in the range 0 to 1, with values of <0.05 considered deleterious, and an estimate of confidence in the assigned score. In using SIFT annotations we excluded any sites tagged as low confidence.

The resulting annotation allowed coding SNPs to be divided into four categories of predicted functional impact: a) synonymous, causing no change in the coded amino acid; b) missense tolerated, amino acid substitutions predicted not to impact protein function based on patterns of protein conservation; c) missense deleterious, amino acid substitutions predicted to disrupt protein function based on patterns of protein conservation; d) nonsense, the introduction of premature stop codons. Per-individual counts of SNPs in each category were used as a relative metric of mutation load, avoiding the need to make assumptions about the average dominance, which would be required in attempting to translate variant counts into explicit estimates of fitness effects. Differences in individual sequencing coverage and overall genetic diversity across populations were accounted for by calculating, per individual, the ratio of variants in a focal category to the number of synonymous variants observed in the same individual.

Separately for autosomal nonsense, missense deleterious, and missense tolerated variant categories, we fitted GLMMs [lme4 with additional statistics from lmerTest; (55)] in which the response variable was the ratio of the number of variants of the focal category/the number of synonymous variants, per individual. Response variables were modeled with a Gaussian error distribution, and model fit was assessed using simulated residuals from DHARMa (56). Initial models included sexual selection regime, sex, and their interaction as fixed effects, with the replicate line ID and individual ID as nested random factors. Interactions were removed if nonsignificant. Pairwise comparisons were performed post hoc using emmeans (57). Models of the same structure were fitted to test the same variant categories on the X chromosome.

We additionally calculated the population-genetic statistic R_xy_ as a complementary measure of relative deleterious variant enrichment between treatments. Details of the analysis and results are provided in *SI Appendix*.

### The Effect of Total Load on Survival Under Inbreeding.

We reanalyzed survival data collected from generation 54 of our experimental populations by Lumley et al. (18) to test whether the differential survival under inbreeding observed in this study is more closely associated with our estimate of mutation load or with the original line-level classification of sexual selection regime. These data track the survival of lineages through multiple generations of enforced sib x sib inbreeding (details in ref. 18). We fitted Cox proportional hazards mixed effects models [coxme; (58)] to survival data from Lumley et al. (18), where survival time was defined as the number of generations to extinction, with surviving populations treated as right-censored. Three models were fitted, each with survival time as the dependent variable, and replicate line within the selection regime as a random factor. In each case, the proportional hazards assumption was verified with the cox.ph function. Independent fixed factors in each model were 1) sexual selection regime, 2) mutation load proxied by counts of deleterious sites, and 3) sexual selection regime + mutation load.

Using our mutation load estimate, we calculated a metric representing the total effect of deleterious variants that are on average partially recessive (59, 60), where variants predicted as nonsense are assigned as “more deleterious” than those predicted to be missense deleterious (as indicated by our mutation load analysis, see *Results*):

$$\mathrm{ML}\;=\;0.25({N_{x}}^{\mathrm{del}\_\mathrm{Het}}\;+\;2.{N_{x}}^{\mathrm{nonsense}\_\mathrm{Het}})\;+\;{N_{x}}^{\mathrm{del}\_\mathrm{Hom}}\;+\;2.{N_{x}}^{\mathrm{nonsense}\_\mathrm{Hom}},$$

where N_x_ represents the ratio of the number of variants of a focal category to the number of synonymous variants observed in the same individual, *del* and *nonsense* refer to missense deleterious and nonsense categories of variants, and *Het* and *Hom* refer to variants in heterozygous and homozygous form respectively. To test the sensitivity of results to this parameter, we repeated the analysis across nine combinations of mean dominance and relative weighting of missense deleterious and nonsense variant effects (*SI Appendix*, *Supplementary Methods*). Results were qualitatively equivalent across load metrics. We therefore present the original case here with further detail provided in *SI Appendix*.

### Assessing Genomic Variation.

Genome-wide nucleotide diversity (π) was computed using pixy v2.0.0 (61) from the allsites vcf containing both variant and invariant sites to avoid upward bias in π estimates. π was computed in nonoverlapping 10 kb windows across all linkage-group chromosomes. Per-population genome-wide π was subsequently aggregated across windows as a weighted mean, using the number of callable comparisons per window as the weight, following the aggregation method recommended in the pixy documentation.

As a proxy for inbreeding in experimental populations, we quantified runs of homozygosity (ROH) using plink v1.9 (52). Analyses were restricted to autosomal linkage groups, and ROH were called using the following parameters: a minimum length of 200 kb (--homozyg-kb 200) and at least 50 SNPs per ROH (--homozyg-snp 50), with a minimum SNP density of one SNP per 25 kb (--homozyg-density 25). Gaps of <100 kb were allowed between consecutive homozygous SNPs within a ROH (*--homozyg-gap 100*). A sliding window approach was applied with 50 SNPs per window (--homozyg-window-snp 50), allowing up to 1 heterozygous call (--homozyg-window-het 1) and 3 missing calls (--homozyg-window-missing 3) per window. A window was required to be called homozygous in at least 5% of its positions to contribute to an ROH (--homozyg-window-threshold 0.05). These parameters largely follow PLINK defaults and those used in analyses of insects and other organisms. The genomic inbreeding coefficient (F_ROH_) was computed, per individual, as the proportion of that individual’s genome in ROH (“KB” field returned by plink divided by the combined callable length of all autosomes). Differences in F_ROH_ between treatments were assessed using the nonparametric Wilcoxon rank-sum test, as the data were right-skewed and could not be transformed to fit a normal distribution.

### Regions of Strong Divergence.

We performed a genome-wide scan for selection using BayPass v2.4 (62), implementing a Bayesian framework sensitive to demography. BayPass estimates a background allele frequency (omega) matrix across populations, to account for the confounding effect of demography which can frustrate the identification of selected variants (62, 63). This approach allowed us to control for unquantified differences in relatedness among individuals used to found each selection line. The BayPass model computes an omega matrix to correct for neutral correlations when testing allele frequencies for population divergence or association with environmental/trait variables. Within BayPass, we utilized the statistic C2, which contrasts SNP allele frequencies between two groups of populations specified by a binary trait. This method outperforms others in identifying SNPs under selection in scenarios analogous to our experiment (29).

We computed C2 across the two sets of three replicate sexual selection lines, with sexual selection regime as the binary covariable. To avoid the impact of small, annotation-sparse, unplaced scaffolds in the reference genome, we ran BayPass on the 10 linkage-group-level scaffolds. We precomputed the BayPass omega matrix using a curated subset of independent, high confidence, highly representative, putatively neutral SNPs which afforded the best opportunity to estimate the neutral covariance in allele frequencies across the genome (omega dataset; see *SI Appendix*, *Supplementary Methods*). BayPass was run independently for autosomal and X-linked loci to reflect their different modes of inheritance. For autosomal runs, the omega dataset consisted of 7,158 nonexonic SNPs, visually confirmed to be evenly spread across the genome. Omega dataset for the X chromosome consisted of 820 SNPs. The dataset used in autosomal BayPass analysis runs contained a less stringently filtered set of 1,833,626 sites, derived from the SNP vcf (see *SI Appendix*, *Supplementary Methods*; 26,259 for the x-linked analysis run).

For each analysis, we performed two independent BayPass runs with different random seed initiators and computed correlations to test the consistency of model performance with our data (29, 64). The C2 estimates were calibrated using a pseudo-observed dataset [POD; (62); see *SI Appendix*, *Supplementary Methods*). The 0.999 quantile of C2 values from the POD analysis was used as the outlier threshold above which to identify C2 candidate SNPs. C2 candidate regions were defined as those containing ≥2 outlier SNPs separated by <50 kb (62), with top candidate SNPs being those in each candidate region with the highest value of C2.

### Effects of BayPass Outliers.

To assess the effects of BayPass outliers on phenotype, we collected functional annotations of coding genes in proximity to these loci. First, we concatenated BayPass outliers identified on the autosomal and X linkage groups, then derived two lists of variants of interest: a) all SNPs with significant C2 (C2 candidate SNPs), to examine divergent regions; b) the SNP with highest C2 from each candidate region (top candidate SNPs), to examine sites more likely to be driving divergence. From each of these lists, a gene set was generated by intersecting their positions with the genome annotation (Tribolium_castaneum.T.cas5.2.59.gff3), one containing the closest gene to each C2 candidate (bedtools -closest), and one containing all genes within 10 kb of a top candidate SNP (bedtools -slop, -intersect). We used these gene lists as input to g:Profiler (30) to test for enrichment of functional terms derived from gene ontology (GO). All other settings were the G:profiler defaults, the background used was all genes in the *T. castaneum* genome. The OSG3 annotation (https://ibeetle-base.uni-goettingen.de/download/species/Tcas/OGS3.gff.gz) and the iBeetle-Base database (65) were used to manually identify gene functions, and orthologous genes in *D.* *melanogaster* were subsequently characterized using Flybase (66).

### Association Between Highly Divergent Regions and Deleterious Variants.

To test whether divergence in candidate regions was driven by purging of deleterious variants, we asked whether highly divergent genomic regions (BayPass C2 outliers) were enriched or depleted for variants predicted to be deleterious (missense deleterious or nonsense) and purged under polyandry (i.e., observed at lower frequency under polyandry relative to monandry). Only outlier regions overlapping coding sequences were included, as some fell entirely in noncoding regions and could not contain annotated variants. For each deleterious variant subset, we compared the observed overlap with a null expectation generated by randomly sampling an equal number of synonymous variants 10,000 times. For each permutation, we counted the number of sampled variants overlapping the outlier regions, producing a null distribution of expected overlap. We calculated an empirical p-value as the proportion of permutations in which the overlap equaled or exceeded the observed value, and a z-score to quantify deviation from the null expectation.

## Supplementary Material

Appendix 01 (PDF)

## Data Availability

Genomic sequences data have been deposited in ENA (ERR15115894-ERR15116070). Previously published data were used for this work (18).
