# Supplementary material for: Sexual selection purges mutation load, but not overall genetic diversity, decreasing vulnerability to extinction
Source: Proc Natl Acad Sci U S A. 2026 Sep 8;123(37):e2608675123. doi: 10.1073/pnas.2608675123 (PMC13578823; doi:10.1073/pnas.2608675123)
Supplement: Supplementary file 1 [file pnas.2608675123.sapp.pdf]

Supplementary methods

Population-level deleterious load with R_{xy}

We also evaluated mutation load at the population level. For this analysis, individuals from replicate lines within a treatment were pooled and treated as a single treatment-level population. Using only coding SNPs, for each category of variant impact, at each site *i* we computed the observed allele frequency of the alternative allele in a population X as $f^{x}_{i} = d^{X}_{i} / n^{X}_{i}$, where n^{X}_{i} is the total number of alleles called at *i* in population X and d^{X}_{i} is the number of alternative alleles called. We computed R_{xy} (described by 1; after the method of 2) for each functional category between monandrous and polyandrous populations, to assess the relative frequency of deleterious variants under each sexual selection regime. Analyses were restricted to sites that were polymorphic in at least one of the compared populations, thereby capturing variants that may have been completely purged from one group. Each R_{xy} value was standardised using the same statistic computed on a random subset of intergenic variants from the same populations. The variance in R_{xy} was estimated by block jackknifing across the data, systematically removing non-overlapping blocks of SNPs 10% of the size of the data (2).

The effect of mutation load on survival under inbreeding

Time to extinction under inbreeding was modelled with a range of different metrics of mutation load, corresponding to different assumptions about the mean dominance and relative weighting of missense deleterious and nonsense variant effects (table S1).

Table S1. Combinations of dominance and weighting of missense deleterious:nonsense variant effects used to compute nine metrics of mutation load.

	Mean dominance of alt allele (h)		
	Totally recessive (h = 0)	Partially recessive (h = 0.25)	Codominant (h = 0.5)
Relative weight (nonsense / missense deleterious; w)	w = 1, h = 0	w = 1, h = 0.25	w = 1, h = 0.5
	w = 2, h = 0	w = 2, h = 0.25	w = 2, h = 0.5
	w = 5, h = 0	w = 5, h = 0.25	w = 5, h = 0.5

The metric of mutation load in each case was computed as:

$$ML = h(N_x^{del\_Het} + w.N_x^{nonsense\_Het}) + N_x^{del\_Hom} + w.N_x^{nonsense\_Hom}$$

Where N_x represents the ratio of the number of variants of a focal category to the number of synonymous variants observed in the same individual, *del* and *nonsense* refer to missense

deleterious and nonsense categories of variants, and *Het* and *Hom* refer to variants in heterozygous and homozygous form respectively.

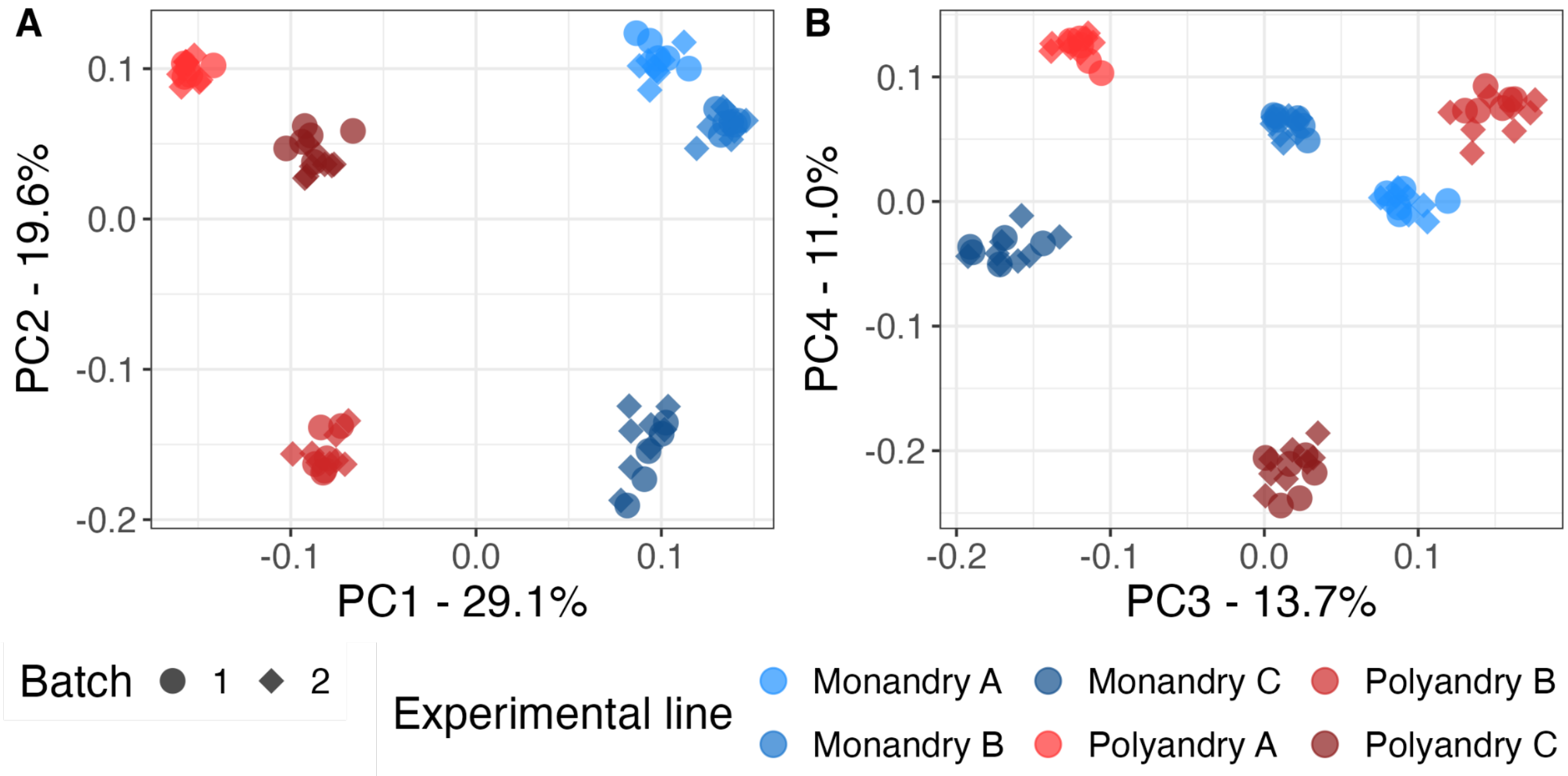

Figure S1. No batch effect from sequencing runs is evident in the data
Principle components summarizing genomic variation of *T. castaneum* populations (colour shade) experimentally evolved under monandrous and polyandrous sexual selection regimes (blue/red). The shape of points indicates in which of two batches the samples were sequenced. No batch effect is evident. The most explanatory principal component (PC1) appears to capture the difference between sexual selection regimes.

Supplementary results

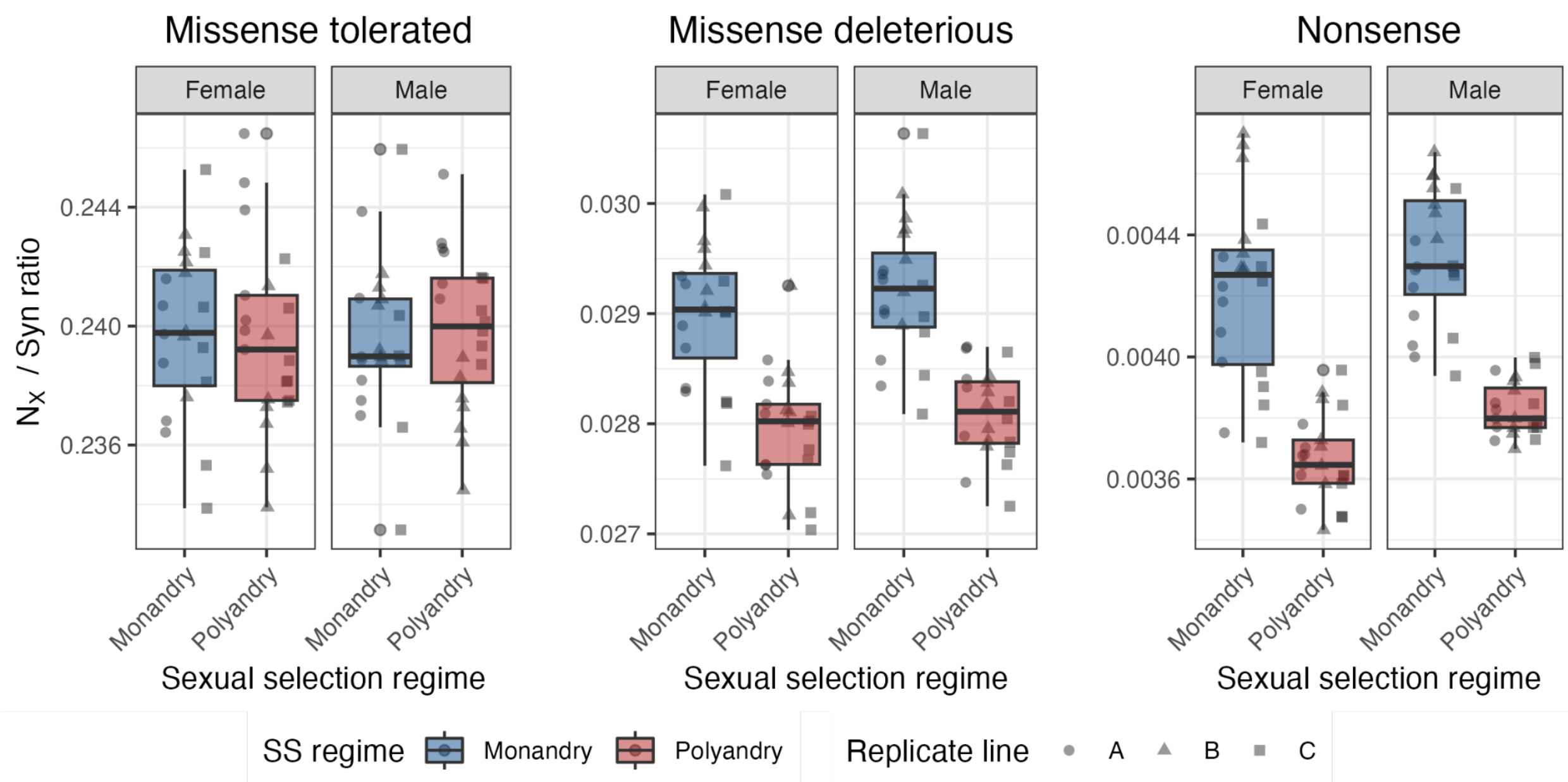

Figure S2. The burden of deleterious variants in the genomes of males and females. The effect of sexual selection on individual mutation load of males and females on the autosomes. The ratio of the number missense tolerated, missense deleterious and nonsense (N_X) variants to the number of synonymous variants per individual, for populations of *Tribolium castaneum* evolving under either monandrous or polyandrous sexual selection regimes for 156 generations. Plots for each functional category are stratified by sex.

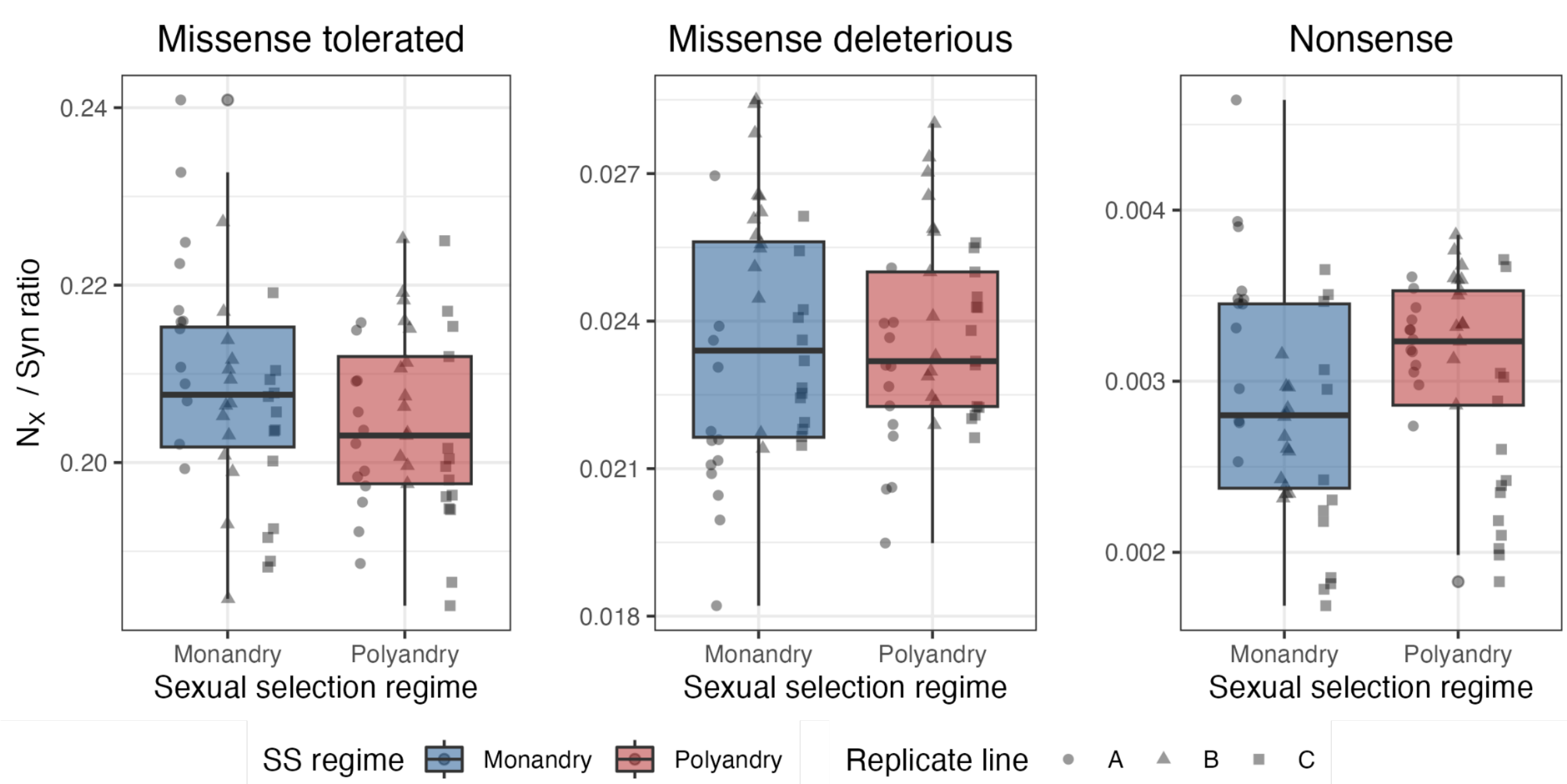

Figure S3. Mutation load on the X chromosome. Genetic variation on the X chromosome categorized by increasing deleterious functional impact (missense tolerated - nonsense) in populations of Tribolium castaneum evolved under either monandrous or polyandrous sexual

selection regimes for 156 generations. Points show the ratio of missense tolerated, missense deleterious and nonsense (NX) sites to synonymous sites, per individual.

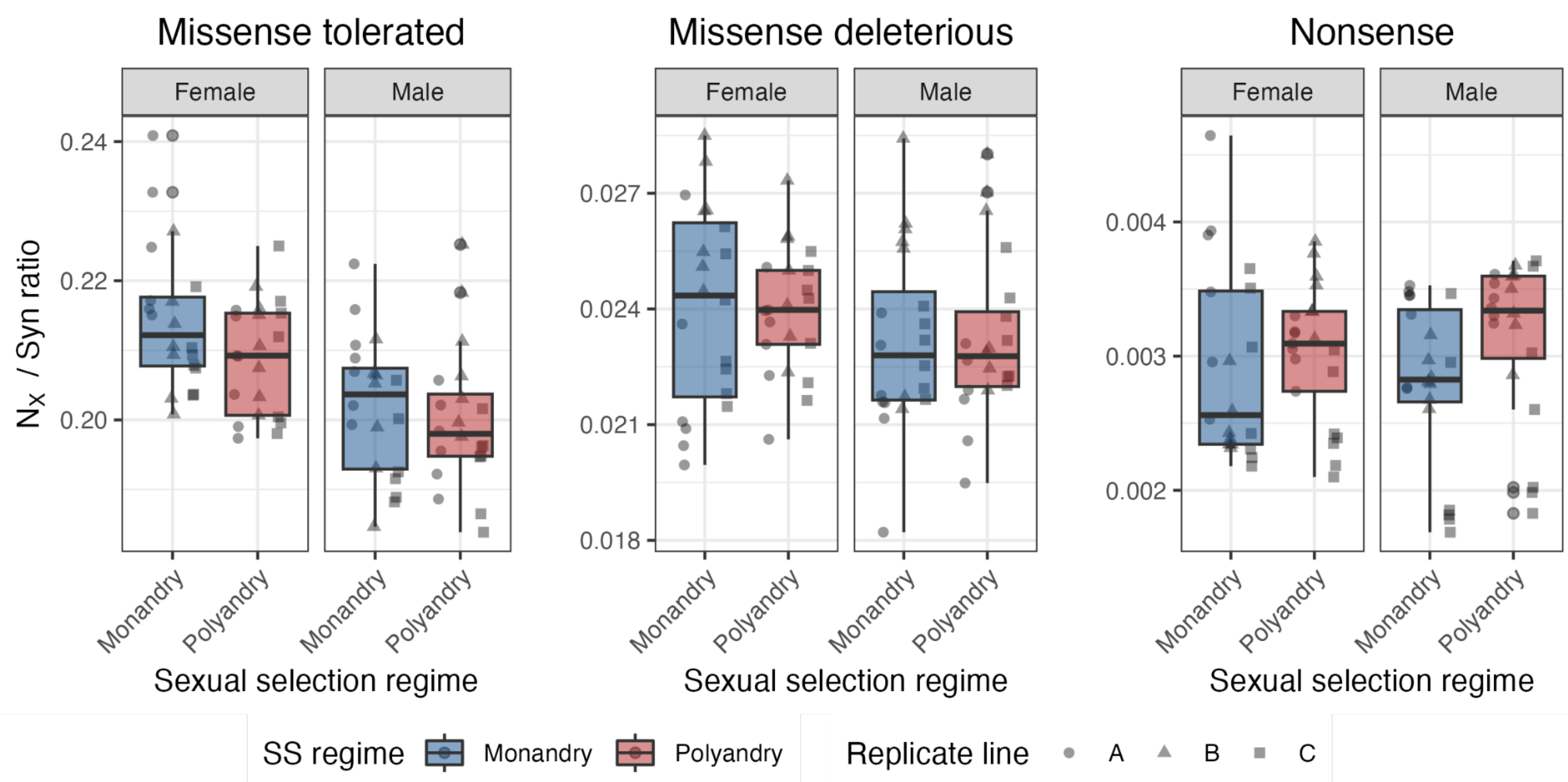

Figure S4. Mutation load on the X chromosome by sex.
The effect of sexual selection on individual mutation load of males and females on the X chromosome. The ratio of the number missense tolerated, missense deleterious and nonsense (NX) variants to the number of synonymous variants per individual, for populations of Tribolium castaneum evolving under either monandrous or polyandrous sexual selection regimes for 156 generations. Plots for each functional category are stratified by sex.

Deleterious autosomal variants are underrepresented in polyandrous populations
To evaluate mutation load at population level, we compared the frequency of missense tolerated, missense deleterious, and nonsense variants between sexual selection regimes. For this analysis line replicates within each regime were pooled to give two ‘populations’ to contrast, to obtain stable estimates of allele frequencies for each functional class and to characterize regime-level differences in the genomic distribution of functional variants. We computed R_{xy} (described by 1; after the method of 2) for each functional category between monandrous and polyandrous populations, where values <1 indicate that variants of the focal category are underrepresented in polyandrous populations relative to monandrous populations. Missense tolerated R_{xy} confidence intervals (CIs) crossed one, indicating no mean difference in frequency between sexual selection regimes (figure 1B). Missense deleterious variants both showed R_{xy} values close to one but with CIs not including one, suggesting a small deficit of these variants under polyandry. Nonsense variants had the lowest values of R_{xy} (jackknife mean = 0.77) with CIs excluding one, indicating that variants with highly deleterious functional impact are strongly underrepresented in genomes from the polyandrous regime (figure 1B). The pattern of R_{xy} across functional categories observed on the autosomes was not replicated on the X chromosome (figure S5). We found no evidence for a relationship between functional

impact and purging strength on the X chromosome, though our ability to detect an effect was likely impacted by small numbers of variants on the X, especially in high impact categories.

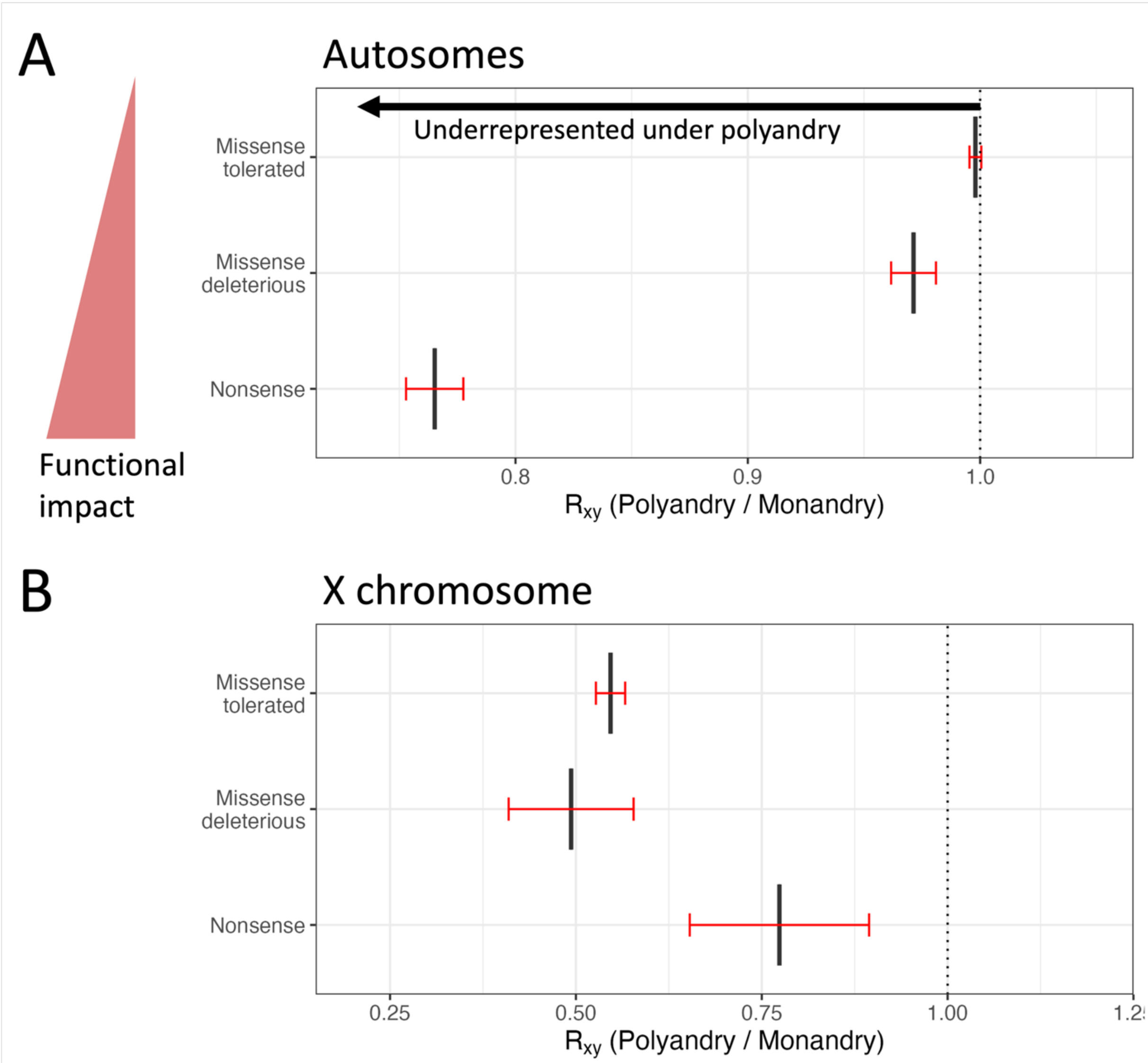

Figure S5. Rxy shows purging of deleterious variants on the autosomes but not the X chromsome

Genetic variation categorised by increasing deleterious functional impact in populations of Tribolium castaneum evolved under either monandrous or polyandrous sexual selection regimes for 156 generations. R_{xy} analysis contrasting allele frequencies in monandrous and polyandrous populations, across functional categories of high-quality SNPs, normalized by Rxy of intergenic variants on A) autosomes and B) the X chromosome. $R_{xy} < 1$ indicates a relative frequency deficit of the corresponding category under polyandry. Red bars represent 95% CIs computed from block jack-knife standard errors.

Mutation load and survival under inbreeding

We compared ability of SS regime and mutation load to predict extinction risk. The main text presents results from the scenario we thought most likely to represent biological reality (core scenario), but to assess the robustness of the result to the load metric used, we repeated the analysis a total of nine metrics of mutation load representing different assumptions about the mean dominance of variants (h) and the relative mean strengths of nonsense variants to missense deleterious and variant effects (w; see supplementary methods).

In the core scenario, extinction time was best predicted by mutation load, with the mutation load model having an AIC at least two less than the SS regime model. This same pattern was observed across every mutation load metric tested, except when h = 0 & w = 5, corresponding to a scenario where deleterious variants are totally recessive and nonsense variants are on average 5 times more deleterious than missense deleterious ones (table S2).
In models fitting both SS regime and mutation load together as fixed factors, SS regime was never a significant predictor of time to extinction. Mutation load remained marginally significant <0.069) in eight of the nine models but its significance dropped relative to being fit alone. This is expected given that we know SS regime and mutation load to be highly colinear.

Overall, the result that mutation load best explained extinction risk was extremely robust to alternative methods of converting counts of variants to estimates of realised mutation load. Full model outputs and fit comparisons are given in table S2.

Table S2. Functional characterisation of genomic variants was used as an estimate of mutation load in 84 genomes sequenced from *Tribolium* castaneum lines evolved under strong (polyandry) or weak (monandry) sexual selection (SS) regimes for 156 generations. Mixed models (GLMM) were fitted to population survival data, when families derived from these same lines where subjected to enforced inbreeding (from 3), modelling survival as a product of mutation load or SS regime or both. The main text shows that the mutation load estimate alone was the best predictor of survival. To evaluate the sensitivity of this result to assumptions, we computed the metric in nine different ways, corresponding to different assumptions about the mean dominance and relative weighting of missense deleterious and nonsense variant effects. **A)** The agreement between models using different metrics of mutation load. **B)** Model comparison using AIC.

A

**Load metric**	**Model**	**SS regime effect (coef)**	**Mutation load effect (coef)**	**Random effect (rep: sd)**
na	SS regime	-0.730, p = 0.026	–	0.020

h = 0, w = 1	Mutation load	–	719.3, p = 0.005	0.009
	SS regime + mutation load	-0.035, p = 0.944	699.0, p = 0.069	0.009
h = 0.25, w = 1	Mutation load	–	378.9, p = 0.003	0.009
	SS regime + mutation load	-0.002, p = 0.997	378.4, p = 0.042	0.009
h = 0.5, w = 1	Mutation load	–	236.2, p = 0.003	0.009
	SS regime + mutation load	-0.092, p = 0.844	220.4, p = 0.051	0.009
h = 0, w = 2	Mutation load	–	632.6, p = 0.006	0.009
	SS regime + mutation load	0.376, p = 0.56	862.8, p = 0.061	0.009
h = 0.25, w = 2	Mutation load	–	299.5, p = 0.0029	0.009
	SS regime + mutation load	0.065, p = 0.900	314.8, p = 0.046	0.009
h = 0.5, w = 2	Mutation load	–	183.3, p = 0.003	0.009
	SS regime + mutation load	-0.071, p = 0.88	173.6, p = 0.054	0.009
h = 0, w = 5	Mutation load	–	331.8, p = 0.020	0.011
	SS regime + mutation load	0.139, p = 0.906	390.1, p = 0.449	0.011
h = 0.25, w = 5	Mutation load	–	109.1, p = 0.003	0.009
	SS regime + mutation load	-0.047, p = 0.925	105.2, p = 0.059	0.009
h = 0.5, w = 5	Mutation load	–	109.1, p = 0.003	0.009
	SS regime + mutation load	-0.047, p = 0.925	1.05.2, p = 0.059	0.009

B

Load metric	Model	LokLik	AIC	ΔAIC from best model	AIC weight
h = 0, w = 1	SS regime	-138.1	280.2	3.13	0.132
	Mutation load	-136.5	277.1	–	0.634
	SS regime + mutation load	-136.5	279.1	2.00	0.234
h = 0.25, w = 1	SS regime	-138.1	280.2	4.071	0.087
	Mutation load	-136.1	276.1	–	0.667
	SS regime + mutation load	-136.1	278.1	2.00	0.245
h = 0.5, w = 1	SS regime	-138.1	280.2	3.722	0.102
	Mutation load	-136.2	276.5	–	0.653
	SS regime + mutation load	-136.2	278.4	1.961	0.245
h = 0, w = 2	SS regime	-138.1	280.2	4.950	0.055
	Mutation load	-136.6	275.3	–	0.661
	SS regime + mutation load	-136.5	276.9	1.675	0.285
h = 0.25, w = 2	SS regime	-138.0	280.0	4.07	0.087
	Mutation load	-136.0	276.0	–	0.667
	SS regime + mutation load	-136.0	278.0	2.00	0.245
h = 0.5, w = 2	SS regime	-138.1	280.2	3.898	0.094
	Mutation load	-136.3	276.3	–	0.661
	SS regime + mutation load	-136.3	278.3	1.984	0.245
h = 0, w = 5	SS regime	-138.1	280.2	0.566	0.355

	Mutation load	-137.8	278.6	–	0.471
	SS regime + mutation load	-137.8	281.6	1.986	0.174
h = 0.25, w = 5	SS regime	-138.1	280.2	3.490	0.113
	Mutation load	-136.4	276.7	–	0.648
	SS regime + mutation load	-136.4	278.7	1.991	0.239
h = 0.5, w = 5	SS regime	-138.1	280.2	3.490	0.113
	Mutation load	-136.4	276.7	–	0.648
	SS regime + mutation load	-136.4	278.7	1.991	0.239

Table S3. Bypass outlier SNPs.

The 442 SNPs identified as significantly associated with sexual selection regime using BayPass. The closest gene to each SNP, the distance and the genomic feature and gene description were obtained by intersecting SNP positions with the genome annotation (Tribolium_castaneum.Tcas5.2.59.gff3). Drosophila homologs were identified with ibeetlebase and characterised with FlyBase.

Chrom	SNP pos	Closest gene	Distance	Feature	Description from T.cast annotation	Fly homolog ID	Fly homolog name
LG10	2875218	TC001520	744	-	-	-	-
LG10	2876118	TC001520	1644	-	-	-	-
LG10	2888791	TC001531	0	intron	-	-	-
LG10	2890963	TC001531	0	intron	-	-	-
LG10	2899728	TC001531	0	intron	-	-	-
LG10	2899752	TC001531	0	intron	-	-	-
LG10	3148149	TC034902	9	intron	UPF0729 protein CG18508-like protein	FBgn0028746	CG18508
LG10	3148218	TC034902	0	3` UTR	"	"	"
LG10	3148505	TC034902	0	5' UTR	"	"	"
LG10	3148505	TC011182	0	exon	60S ribosomal protein L7a-like Protein	FBgn0014026	Ribosomal protein L7A
LG10	3148524	TC034902	0	5' UTR	UPF0729 protein CG18508-like protein	FBgn0028746	CG18508
LG10	3148524	TC011182	0	intron	60S ribosomal protein L7a-like Protein	FBgn0014026	Ribosomal protein L7A
LG10	3148657	TC011182	0	intron	"	"	"
LG10	3148712	TC011182	0	intron	"	"	"
LG10	3148988	TC011182	0	exon	"	"	"
LG10	3149014	TC011182	0	exon	"	"	"
LG10	3149472	TC011182	0	intron	"	"	"
LG10	3149481	TC011182	0	intron	"	"	"
LG10	3149493	TC011182	0	intron	"	"	"
LG10	3150585	TC011181	0	exon	Serine/threonine-protein phosphatase	FBgn0003139	Protein phosphatase V
LG10	3151854	TC011310	0	exon	Pre-mRNA-processing factor 17-like Protein	FBgn0038927	CG6015
LG10	3151998	TC011310	0	exon	"	"	"
LG10	3152469	TC011310	0	intron	"	"	"

LG10	3152507	TC011310	0	intron	"	"	"
LG10	3153195	TC011310	0	exon	"	"	"
LG2	6360635	TC000829	0	intron	Serine protease H18	FBgn0035496	Serine protease homolog (var)
LG2	6360646	TC000829	0	intron	"	"	"
LG2	6361627	TC000829	0	exon	"	"	"
LG2	6361627	TC030253	0	intron	Gustatory receptor	-	-
LG2	6361719	TC000829	0	exon	Serine protease H18	FBgn0035496	Serine protease homolog 35
LG2	6361719	TC030253	0	intron	Gustatory receptor	-	-
LG2	6361730	TC000829	0	exon	Serine protease H18	FBgn0035496	Serine protease homolog 35
LG2	6361730	TC030253	0	intron	Gustatory receptor	-	-
LG2	6361884	TC000829	0	exon	Serine protease H18	FBgn0035496	Serine protease homolog 35
LG2	6361884	TC030253	0	intron	Gustatory receptor	-	-
LG2	6362638	TC000829	0	intron	Serine protease H18	FBgn0035496	Serine protease homolog 35
LG2	6363263	TC000829	0	intron	"	"	"
LG2	6363541	TC000829	0	intron	"	"	"
LG2	6363803	TC000829	0	intron	"	"	"
LG2	6363874	TC000829	0	intron	"	"	"
LG2	6364637	TC000829	0	intron	"	"	"
LG2	6364854	TC000829	0	intron	"	"	"
LG2	6364859	TC000829	0	intron	"	"	"
LG2	6366349	TC000829	298	-	"	"	"
LG2	6366418	TC000829	367	-	"	"	"
LG2	6367320	TC000829	1269	-	"	"	"
LG2	6367492	TC000829	1441	-	"	"	"
LG2	6367750	TC000829	1699	-	"	"	"

LG2	6367753	TC000829	1702	-	"	"	"
LG2	6367763	TC000829	1712	-	"	"	"
LG2	6367974	TC000829	1923	-	"	"	"
LG2	6368123	TC000829	2072	-	"	"	"
LG2	6368173	TC000829	2122	-	"	"	"
LG2	6369222	TC000829	3171	-	"	"	"
LG2	6369242	TC000829	3191	-	"	"	"
LG2	6369280	TC000829	3229	-	"	"	"
LG2	6371431	TC000829	5380	-	"	"	"
LG2	6371729	TC000829	5678	-	"	"	"
LG2	6371733	TC000829	5682	-	"	"	"
LG2	6371741	TC000829	5690	-	"	"	"
LG2	6371746	TC000829	5695	-	"	"	"
LG2	6371749	TC000829	5698	-	"	"	"
LG2	6372049	TC000829	5998	-	"	"	"
LG2	6372237	TC000829	6186	-	"	"	"
LG2	6372297	TC000829	6246	-	"	"	"
LG2	6373869	TC000829	7818	-	"	"	"
LG2	6759639	TC032076	0	intron	Disks large 1 tumor suppressor protein-like Protein	FBgn0001624	discs large 1
LG2	6759675	TC032076	0	intron	"	"	"
LG2	6767618	TC032076	0	intron	"	"	"
LG2	12685100	TC001207	0	intron	RNA-binding protein MEX3B-like Protein	FBgn0039920	CG11360
LG2	12695008	TC032210	1005	-	-	-	-
LG2	12695027	TC032210	1024	-	-	-	-
LG2	12695032	TC032210	1029	-	-	-	-
LG2	12696786	TC000351	1162	-	-	-	-
LG2	12708148	TC000351	0	intron	-	-	-

LG2	12710288	TC000351	0	intron	-	-	-
LG2	12710438	TC000351	0	intron	-	-	-
LG2	12712223	TC000351	0	intron	-	-	-
LG3	25378890	TC034629	0	intron	Hepatic leukemia factor-like protein	FBgn0016694	PAR-domain protein 1
LG3	25383208	TC034629	0	intron	"	"	"
LG3	25385605	TC034629	0	intron	"	"	"
LG3	25386766	TC034629	0	intron	"	"	"
LG4	9239595	TC008372	0	intron	Leucine--tRNA ligase%2C cytoplasmic-like Protein	FBgn0284253	Leucyl-tRNA synthetase
LG4	9239957	TC008372	0	intron	"	"	"
LG4	9240001	TC008372	0	intron	"	"	"
LG4	9240050	TC008372	0	intron	"	"	"
LG4	9240163	TC008372	0	intron	"	"	"
LG4	9240232	TC008372	0	intron	"	"	"
LG4	9240233	TC008372	0	intron	"	"	"
LG4	9240250	TC008372	0	intron	"	"	"
LG4	9240287	TC008372	0	intron	"	"	"
LG4	9240308	TC008372	0	intron	"	"	"
LG4	9240313	TC008372	0	intron	"	"	"
LG4	9240325	TC008372	0	intron	"	"	"
LG4	9240394	TC008372	0	intron	"	"	"
LG4	9248921	TC007110	1542	-	-	FBgn0031016	kekkon 5
LG4	9248923	TC007110	1540	-	"	"	"
LG4	9248925	TC007110	1538	-	"	"	"
LG6	9546142	TC014863	2200	-	43 kDa receptor-associated protein of the synapse homolog-like Protein	FBgn0039911	CG1909
LG6	9620940	TC014861	0	exon	Neuroblastoma%2C suppression of tumorigenicity 1	-	-
LG6	9621142	TC014861	0	exon	"	-	-

LG6	9623478	TC014861	0	intron	"	-	-
LG6	9623731	TC014861	0	exon	"	-	-
LG6	9625178	TC014861	0	intron	"	-	-
LG6	9632402	TC014860	0	intron	Moesin/ezrin/radixin homolog 2-like Protein	FBgn0086384	Merlin
LG6	9632454	TC014860	0	intron	"	"	"
LG6	9640445	TC014859	0	intron	Zinc finger HIT domain-containing protein 2-like Protein	FBgn0051223	CG31223
LG6	9640628	TC014859	0	intron	"	"	"
LG6	9641505	TC014859	0	intron	"	"	"
LG6	9641518	TC014859	0	intron	"	"	"
LG6	9647714	TC014857	0	exon	Ribosome biogenesis methyltransferase WBSCR22-like Protein	FBgn0037543	CG10903
LG6	9648497	TC015858	37	-	Cell division control protein 45 homolog-like Protein	FBgn0026143	Cell division cycle 45
LG6	9648756	TC015858	0	exon	"	"	"
LG6	9649654	TC015858	0	exon	"	"	"
LG6	9649716	TC015858	0	intron	"	"	"
LG6	9649822	TC015858	0	intron	"	"	"
LG6	9649865	TC015858	0	intron	"	"	"
LG6	9652242	TC015858	0	exon	"	"	"
LG6	9652269	TC015858	0	exon	"	"	"
LG6	9652392	TC015858	0	exon	"	"	"
LG6	9659272	TC014856	0	intron	-	-	-
LG6	9662151	TC014856	0	exon	-	-	-
LG6	9664520	TC014856	0	intron	-	-	-
LG6	9664534	TC014856	0	intron	-	-	-
LG6	9664921	TC014856	0	intron	-	-	-
LG6	9665270	TC014856	0	intron	-	-	-
LG6	9669740	TC014856	0	intron	-	-	-

LG6	9669776	TC014856	0	intron	-	-	-
LG6	9670076	TC014856	0	intron	-	-	-
LG6	9671781	TC014856	0	intron	-	-	-
LG6	9674940	TC014856	0	intron	-	-	-
LG6	9675271	TC014856	0	intron	-	-	-
LG6	9675299	TC014856	0	intron	-	-	-
LG6	9675442	TC014856	0	intron	-	-	-
LG6	9686696	TC014856	5404	-	-	-	-
LG6	9686813	TC014856	5521	-	-	-	-
LG7	1184860	TC009377	776	-	-	FBgn0083951	CG34115
LG7	1184872	TC009377	764	-	-	"	"
LG7	1184873	TC009377	763	-	-	"	"
LG7	1188869	TC009441	0	intron	Collagen type IV alpha-3-binding protein-like Protein	FBgn0027569	ceramide transfer protein
LG8	12415127	TC005479	0	intron	Polypeptide N-acetylgalactosaminyltransferase	FBgn0050463	Polypeptide N-Acetylgalactosaminyltr ansferase 9
LG8	12415169	TC005479	0	intron	"	"	"
LG8	12415200	TC005479	0	intron	"	"	"
LG8	12415247	TC005479	0	intron	"	"	"
LG8	12415408	TC005479	0	intron	"	"	"
LG8	12459002	TC006646	2969	-	-	FBgn0038819	Cuticular protein 92F
LG8	12459035	TC006646	2936	-	-	"	"
LG8	12509462	TC005477	3	-	Serine/threonine-protein kinase ULK2-like Protein	FBgn0260945	Autophagy-related 1
LG8	12509497	TC005477	38	-	"	"	"
LG8	12511174	TC034152	0	3` UTR	-	-	-
LG8	12538848	TC034154	508	-	-	-	-
LG8	12539021	TC034154	681	-	-	-	-
LG8	12540431	TC034154	2091	-	-	-	-

LG8	12545606	TC034154	7266	-	-	-	-
LG8	12545612	TC034154	7272	-	-	-	-
LG8	12545951	TC034154	7611	-	-	-	-
LG8	12565644	TC005473	0	intron	-	FBgn0263994	CG43737
LG8	12569527	TC005473	0	intron	-	"	"
LG9	8097869	TC034351	0	exon	-	FBgn0261836	Muscle-specific protein 300 kDa
LG9	8103170	TC034351	0	exon	-	"	"
LG9	8103173	TC034351	0	exon	-	"	"
LG9	8113123	TC034351	0	exon	-	"	"
LG9	8804148	TC034357	18651	-	-	FBgn0287478	Calmodulin-binding transcription activator
LG9	8805772	TC034357	17027	-	-	"	"
LG9	8815857	TC034357	6942	-	-	"	"
LG9	8815958	TC034357	6841	-	-	"	"
LG9	8817105	TC034357	5694	-	-	"	"
LG9	8817652	TC034357	5147	-	-	"	"
LG9	8822755	TC034357	44	-	-	"	"
LG9	8824374	TC034357	0	3`UTR	-	"	"
LG9	8824863	TC034357	0	intron	-	"	"
LG9	8825322	TC034357	0	intron	-	"	"
LG9	8830110	TC034357	0	intron	-	"	"
LG9	8830151	TC034357	0	intron	-	"	"
LG9	8831762	TC034357	0	intron	-	"	"
LG9	8834324	TC034357	0	intron	-	"	"
LG9	8838455	TC034357	0	intron	-	"	"
LG9	8839515	TC034357	0	intron	-	"	"
LG9	8839675	TC034357	0	intron	-	"	"
LG9	8839823	TC034357	0	intron	-	"	"

LG9	8839890	TC034357	0	intron	-	"	"
LG9	8839917	TC034357	0	intron	-	"	"
LG9	8839929	TC034357	0	intron	-	"	"
LG9	8839973	TC034357	0	intron	-	"	"
LG9	8839977	TC034357	0	intron	-	"	"
LG9	8839991	TC034357	0	intron	-	"	"
LG9	8839994	TC034357	0	intron	-	"	"
LG9	8840023	TC034357	0	intron	-	"	"
LG9	8840043	TC034357	0	intron	-	"	"
LG9	8840087	TC034357	0	intron	-	"	"
LG9	8840169	TC034357	0	intron	-	"	"
LG9	8840559	TC034357	0	exon	-	"	"
LG9	8840560	TC034357	0	exon	-	"	"
LG9	8840575	TC034357	0	exon	-	"	"
LG9	8843888	TC034357	0	intron	-	"	"
LG9	8844155	TC034357	0	intron	-	"	"
LG9	8844190	TC034357	0	intron	-	"	"
LG9	8844302	TC034357	0	intron	-	"	"
LG9	8844346	TC034357	0	intron	-	"	"
LG9	8844347	TC034357	0	intron	-	"	"
LG9	8844348	TC034357	0	intron	-	"	"
LG9	8844460	TC034357	0	intron	-	"	"
LG9	8844465	TC034357	0	intron	-	"	"
LG9	8844499	TC034357	0	intron	-	"	"
LG9	8844514	TC034357	0	intron	-	"	"
LG9	8844602	TC034357	0	intron	-	"	"
LG9	8844715	TC034357	0	intron	-	"	"
LG9	8848978	TC034357	0	intron	-	"	"

LG9	8849778	TC034357	0	intron	-	"	"
LG9	8850259	TC034357	0	intron	-	"	"
LG9	8850303	TC034357	0	intron	-	"	"
LG9	8851956	TC034357	0	intron	-	"	"
LG9	8853079	TC034357	0	intron	-	"	"
LG9	8853087	TC034357	0	intron	-	"	"
LG9	8853094	TC034357	0	intron	-	"	"
LG9	8871126	TC034357	0	intron	-	"	"
LG9	8871142	TC034357	0	intron	-	"	"
LG9	8872540	TC034357	0	intron	-	"	"
LG9	8872611	TC034357	0	intron	-	"	"
LG9	8872613	TC034357	0	intron	-	"	"
LG9	8872659	TC034357	0	intron	-	"	"
LG9	8881740	TC034357	0	intron	-	"	"
LGX	3211320	TC014210	28	-	Guanylate kinase-like Protein	FBgn0036099	oya
LGX	3211495	TC014210	0	5' UTR	"	"	"
LGX	3211514	TC014210	0	5' UTR	"	"	"
LGX	3211671	TC014210	0	intron	"	"	"
LGX	3212007	TC014210	0	exon	"	"	"
LGX	3212208	TC014210	0	intron	"	"	"
LGX	3212306	TC014210	0	exon	"	"	"
LGX	3212954	TC014210	330	-	"	"	"
LGX	3212987	TC014210	363	-	"	"	"
LGX	3213547	TC013603	330	-	Gamma-glutamylcyclotransferase	FBgn0030411	CG2540
LGX	3213563	TC013603	314	-	"	"	"
LGX	3231380	TC013602	3409	-	Vitellogenin-like Protein	-	-
LGX	3231485	TC013602	3304	-	"	-	-
LGX	3234141	TC013602	648	-	"	-	-

LGX	3234628	TC013602	161	-	"	-	-
LGX	3234672	TC013602	117	-	"	-	-
LGX	3234990	TC013602	0	exon	"	-	-
LGX	3235611	TC013602	0	exon	"	-	-
LGX	3236513	TC013602	0	exon	"	-	-
LGX	3236534	TC013602	0	exon	"	-	-
LGX	3236618	TC013602	0	exon	"	-	-
LGX	3236786	TC013602	0	exon	"	-	-
LGX	3236915	TC013602	0	exon	"	-	-
LGX	3237068	TC013602	0	exon	"	-	-
LGX	3237077	TC013602	0	exon	"	-	-
LGX	3237179	TC013602	0	exon	"	-	-
LGX	3237272	TC013602	0	exon	"	-	-
LGX	3237278	TC013602	0	exon	"	-	-
LGX	3237305	TC013602	0	exon	"	-	-
LGX	3237479	TC013602	0	exon	"	-	-
LGX	3237795	TC013602	0	exon	"	-	-
LGX	3238015	TC013602	0	exon	"	-	-
LGX	3238276	TC013602	0	exon	"	-	-
LGX	3238312	TC013602	0	exon	"	-	-
LGX	3238330	TC013602	0	exon	"	-	-
LGX	3238365	TC013602	0	exon	"	-	-
LGX	3239065	TC013602	0	exon	"	-	-
LGX	3239137	TC013602	0	exon	"	-	-
LGX	3239356	TC013602	0	exon	"	-	-
LGX	3239398	TC013602	0	exon	"	-	-
LGX	3240529	TC013602	43	-	"	-	-
LGX	3241290	TC014211	0	5' UTR	Neuropeptide Y receptor-like Protein	FBgn0038880	SIFamide receptor

LGX	3242080	TC014211	0	intron	"	"	"
LGX	3242093	TC014211	0	intron	"	"	"
LGX	3242120	TC014211	0	intron	"	"	"
LGX	3242820	TC014211	0	intron	"	"	"
LGX	3242897	TC014211	0	exon	"	"	"
LGX	3243399	TC014211	0	intron	"	"	"
LGX	3243456	TC014211	0	intron	"	"	"
LGX	3243543	TC014211	0	intron	"	"	"
LGX	3244121	TC014211	0	exon	"	"	"
LGX	3244294	TC014211	0	exon	"	"	"
LGX	3244641	TC014211	81	-	"	"	"
LGX	3244850	TC014211	290	-	"	"	"
LGX	3245312	TC013601	367	-	Putative DNA mismatch repair protein Msh6-like Protein	FBgn0036486	Msh6
LGX	3248338	TC013601	0	intron	"	"	"
LGX	3248353	TC013601	0	intron	"	"	"
LGX	3248379	TC013601	0	intron	"	"	"
LGX	3248670	TC013601	0	intron	"	"	"
LGX	3248686	TC013601	0	intron	"	"	"
LGX	3248697	TC013601	0	intron	"	"	"
LGX	3248783	TC013601	0	intron	"	"	"
LGX	3248929	TC013601	0	intron	"	"	"
LGX	3256119	TC031503	0	intron	Xaa-Pro dipeptidase-like Protein	FBgn0000455	Dipeptidase C
LGX	3257072	TC031503	0	intron	"	"	"
LGX	3257317	TC031503	0	intron	"	"	"
LGX	3257872	TC031503	0	intron	"	"	"
LGX	3257880	TC031503	0	intron	"	"	"
LGX	3258590	TC031503	0	intron	"	"	"

LGX	3258917	TC031503	0	intron	"	"	"
LGX	3259209	TC031503	0	intron	"	"	"
LGX	3259736	TC031503	0	intron	"	"	"
LGX	3259806	TC031503	0	intron	"	"	"
LGX	3262032	TC031503	0	intron	"	"	"
LGX	3262904	TC031503	0	intron	"	"	"
LGX	3285248	TC030882	0	intron	Arginine kinase-like Protein	FBgn0000116	Arginine kinase 1
LGX	3291587	TC031505	0	intron	-	-	-
LGX	3291733	TC031505	0	intron	-	-	-
LGX	3297041	TC013595	0	5' UTR	-	FBgn0030847	CG12991
LGX	3410074	TC013586	0	intron	-	FBgn0261885	osa
LGX	3410669	TC013586	0	intron	-	"	"
LGX	3410880	TC013586	0	intron	-	"	"
LGX	3410895	TC013586	0	intron	-	"	"
LGX	3411038	TC013586	0	intron	-	"	"
LGX	3411150	TC013586	0	intron	-	"	"
LGX	3412330	TC013586	0	intron	-	"	"
LGX	3413050	TC013586	0	intron	-	"	"
LGX	3413380	TC013586	0	intron	-	"	"
LGX	3413719	TC013586	0	intron	-	"	"
LGX	3413791	TC013586	0	intron	-	"	"
LGX	3413897	TC013586	0	intron	-	"	"
LGX	3414129	TC013586	0	intron	-	"	"
LGX	3414474	TC013586	0	intron	-	"	"
LGX	3416432	TC013586	0	intron	-	"	"
LGX	3416597	TC013586	0	intron	-	"	"
LGX	3416687	TC013586	0	intron	-	"	"
LGX	3417628	TC013586	0	intron	-	"	"

LGX	3417908	TC013586	0	intron	-	"	"
LGX	3417923	TC013586	0	intron	-	"	"
LGX	3418078	TC013586	0	intron	-	"	"
LGX	3418168	TC013586	0	intron	-	"	"
LGX	3418294	TC013586	0	intron	-	"	"
LGX	3418757	TC013586	0	intron	-	"	"
LGX	3418817	TC013586	0	intron	-	"	"
LGX	3418900	TC013586	0	intron	-	"	"
LGX	3419421	TC013586	0	intron	-	"	"
LGX	3419477	TC013586	0	intron	-	"	"
LGX	3419563	TC013586	0	intron	-	"	"
LGX	3421349	TC013586	0	intron	-	"	"
LGX	3421484	TC013586	0	intron	-	"	"
LGX	3421675	TC013586	0	intron	-	"	"
LGX	3422074	TC013586	0	intron	-	"	"
LGX	3422103	TC013586	0	intron	-	"	"
LGX	3422156	TC013586	0	intron	-	"	"
LGX	3422270	TC013586	0	intron	-	"	"
LGX	3424571	TC013586	0	5' UTR	-	"	"
LGX	3425229	TC013585	0	3' UTR	Katanin p60 ATPase-containing subunit A1	FBgn0040208	Katanin 60
LGX	3425246	TC013585	0	3' UTR	"	"	"
LGX	3425252	TC013585	0	3' UTR	"	"	"
LGX	3425366	TC013585	0	3' UTR	"	"	"
LGX	3425615	TC013585	0	exon	"	"	"
LGX	3426178	TC013585	0	exon	"	"	"
LGX	3426416	TC013585	0	exon	"	"	"
LGX	3426671	TC013585	0	intron	"	"	"
LGX	3426782	TC013585	0	exon	"	"	"

LGX	3426791	TC013585	0	exon	"	"	"
LGX	3426927	TC013585	0	intron	"	"	"
LGX	3426989	TC013585	0	exon	"	"	"
LGX	3427004	TC013585	0	exon	"	"	"
LGX	3427037	TC013585	0	exon	"	"	"
LGX	3427266	TC013585	0	-	"	"	"
LGX	3427580	TC013585	28	-	"	"	"
LGX	3427596	TC013585	44	-	"	"	"
LGX	3427610	TC013585	58	-	"	"	"
LGX	3427632	TC013585	80	-	"	"	"
LGX	3427664	TC013585	112	-	"	"	"
LGX	3427704	TC014218	120	-	RNA pseudouridylate synthase domain-containing protein 1-like Protein	-	-
LGX	3427739	TC014218	85	-	"	-	-
LGX	3427745	TC014218	79	-	"	-	-
LGX	3427895	TC014218	0	exon	"	-	-
LGX	3427908	TC014218	0	exon	"	-	-
LGX	3428246	TC014218	0	exon	"	-	-
LGX	3428338	TC014218	0	intron	"	-	-
LGX	3428391	TC014218	0	exon	"	-	-
LGX	3428639	TC014218	0	intron	"	-	-
LGX	3428822	TC014218	0	3' UTR	"	-	-
LGX	3429228	TC014219	0	exon	Protein jagunal-like Protein	FBgn0037374	jagunal
LGX	3429573	TC014219	0	intron	"	"	"
LGX	3429575	TC014219	0	intron	"	"	"
LGX	3429711	TC014219	0	exon	"	"	"
LGX	3429733	TC014219	0	3' UTR	"	"	"
LGX	3429815	TC014219	0	3' UTR	"	"	"

LGX	3430593	TC014220	0	intron	Monocarboxylate transporter 9-like Protein	FBgn0033028	hermes
LGX	3430846	TC014220	0	intron	"	"	"
LGX	3430854	TC014220	0	intron	"	"	"
LGX	3431112	TC014220	0	5' UTR	"	"	"
LGX	3431113	TC014220	0	5' UTR	"	"	"
LGX	3431117	TC014220	0	5' UTR	"	"	"
LGX	3432141	TC014220	0	intron	"	"	"
LGX	3432185	TC014220	0	intron	"	"	"
LGX	3432202	TC014220	0	intron	"	"	"
LGX	3432226	TC014220	0	intron	"	"	"
LGX	3432236	TC014220	0	intron	"	"	"
LGX	3432259	TC014220	0	intron	"	"	"
LGX	3432728	TC014220	0	intron	"	"	"
LGX	3432769	TC014220	0	intron	"	"	"
LGX	3432931	TC014220	0	exon	"	"	"
LGX	3433392	TC014220	0	exon	"	"	"
LGX	3433495	TC014220	0	exon	"	"	"
LGX	3434350	TC014220	0	3' UTR	"	"	"
LGX	3434839	TC014220	116	-	"	"	"
LGX	3435444	TC014221	0	intron	UPF0547 protein C16orf87 homolog-like Protein	-	-
LGX	3435653	TC014221	0	exon	"	-	-
LGX	3435837	TC014221	0	3' UTR	"	-	-
LGX	3435837	TC013584	0	intron	-	-	-
LGX	3435838	TC014221	0	3' UTR	UPF0547 protein C16orf87 homolog-like Protein	-	-
LGX	3435838	TC013584	0	intron	-	-	-
LGX	3435883	TC014221	0	3' UTR	UPF0547 protein C16orf87 homolog-like Protein	-	-

LGX	3435883	TC013584	0	intron	-	-	-
LGX	3435884	TC014221	0	3' UTR	UPF0547 protein C16orf87 homolog-like Protein	-	-
LGX	3435884	TC013584	0	intron	-	-	-
LGX	3435891	TC014221	0	3' UTR	UPF0547 protein C16orf87 homolog-like Protein	-	-
LGX	3435891	TC013584	0	intron	-	-	-
LGX	3436008	TC014221	0	3' UTR	UPF0547 protein C16orf87 homolog-like Protein	-	-
LGX	3436008	TC013584	0	intron	-	-	-
LGX	3436353	TC014222	0	5' UTR	Nischarin-like Protein	FBgn0033996	CG11807
LGX	3436864	TC014222	0	intron	"	"	"
LGX	3437510	TC014222	0	exon	"	"	"
LGX	3437580	TC014222	0	exon	"	"	"
LGX	3437916	TC014222	0	exon	"	"	"
LGX	3438014	TC014222	0	3' UTR	"	"	"
LGX	3438066	TC014222	49	-	"	"	"
LGX	3438498	TC030883	0	5' UTR	-	-	-
LGX	3438602	TC030883	0	intron	-	-	-
LGX	3438812	TC030883	0	5' UTR	-	-	-
LGX	3438871	TC030883	0	exon	-	-	-
LGX	3438873	TC030883	0	exon	-	-	-
LGX	3439103	TC030883	0	intron	-	-	-
LGX	3439637	TC030883	0	intron	-	-	-
LGX	3439666	TC030883	0	intron	-	-	-
LGX	3439708	TC030883	0	intron	-	-	-
LGX	3439777	TC030883	0	intron	-	-	-
LGX	3439851	TC030883	0	intron	-	-	-
LGX	3439955	TC030883	0	intron	-	-	-

LGX	3440721	TC030883	0	exon	-	-	-
LGX	3440934	TC030883	67	-	-	-	-
LGX	3441341	TC030884	32	-	Phosphatidylinositol 3-kinase regulatory subunit alpha-like Protein	FBgn0020622	Pi3K21B
LGX	3444819	TC030884	0	intron	"	"	"
LGX	3444894	TC030884	0	intron	"	"	"
LGX	3445814	TC030884	0	intron	"	"	"
LGX	3446359	TC030884	0	intron	"	"	"
LGX	3449862	TC030884	0	intron	"	"	"
LGX	3449981	TC030884	0	intron	"	"	"
LGX	3453755	TC030884	0	exon	"	"	"
LGX	3456897	TC013582	0	exon	Cathepsin K	FBgn0032228	Cathepsin L4
LGX	3457479	TC013582	0	exon	"		
LGX	3457721	TC014225	0	5'UTR	Transcription factor E3-like Protein	FBgn0263112	Mitf
LGX	3457804	TC014225	0	5' UTR	"	"	"
LGX	3460463	TC014225	0	intron	"	"	"
LGX	3464581	TC014225	0	intron	"	"	"
LGX	3465929	TC014225	0	intron	"	"	"
LGX	3466970	TC014225	0	intron	"	"	"
LGX	3467613	TC014225	0	intron	"	"	"
LGX	3468887	TC014225	0	3' UTR	"	"	"
LGX	3468975	TC014225	0	3' UTR	"	"	"
LGX	3470039	TC014225	925	-	"	"	"
LGX	3470189	TC014225	1075	-	"	"	"
LGX	3470239	TC014225	1125	-	"	"	"
LGX	3470249	TC014225	1135	-	"	"	"
LGX	3471429	TC014226	321	-	Kinesin-like protein KIF13A	FBgn0019968	Kinesin heavy chain 73
LGX	3472717	TC014226	0	intron	"	"	"

LGX	3473260	TC014226	0	intron	"	"	"

Table S4. Genes from significantly divergent regions. Genes identified in regions divergent between *Tribolium castaneum* individuals evolved under either strong or weak sexual selection. The genome scan for divergence was performed with BayPass between three polyandrous and three monandrous populations evolved under these regimes for 156 generations. Gene functions were obtained from Tcas5.2 genome annotation and cross-referenced with FlyBase via the iBeetlebase database.

Chrom	SNP pos	Gene	Description from T.cast annotation	Fly homolog ID	Fly homolog name	Fly homolog function	Reference
LG10	2899728	TC001531	-	-	-	-	-
LG10	3148712	TC011184	UPF0668 protein C10orf76 homolog-like Protein	FBgn0037638	CG8379	-	-
LG10	3148712	TC011183	mRNA turnover protein 4 homolog-like Protein	FBgn0033485	Ribosomal protein LP0-like	cytoplasmic translation	4
LG10	3148712	TC034902	UPF0729 protein CG18508-like protein	FBgn0028746	CG18508	-	-
LG10	3148712	TC011182	60S ribosomal protein L7a-like Protein	FBgn0014026	Ribosomal protein L7A	cytoplasmic translation	4
LG10	3148712	TC011181	Serine/threonine-protein phosphatase	FBgn0003139	Protein phosphatase V	mitotic cell cycle	5
LG10	3148712	TC011310	Pre-mRNA-processing factor 17-like Protein	FBgn0038927	CG6015	mRNA splicing; spermatogenesis	6
LG10	3148712	TC011311	Homeobox protein extradenticle-like Protein	FBgn0000611	extradenticle	brain / peripheral nervous system development	7
LG10	3148712	TC011312	Rab GTPase-activating protein 1-like Protein	FBgn0035879	GTPase activating protein and centrosome-associated	-	-
LG10	3148712	TC011309	-	FBgn0260789	Multi sex combs	hemocyte differentiation	8
LG2	6361719	TC000745	DNA damage-regulated autophagy modulator protein	FBgn0025624	CG4025	autophagy regulation	-

			2-like Protein				
LG2	6361719	TC030253	Gustatory receptor	-	-	-	-
LG2	6361719	TC000829	Serine protease H18	FBgn0032213	Serine protease homolog 35	peptidase activity	9
LG2	6767618	TC032076	Disks large 1 tumor suppressor protein-like Protein	FBgn0001624	discs large 1	various, inc: locomotor rhythm; male courtship beh, mating beh	10
LG2	12710438	TC000351	-	-	-	-	-
LG2	12710438	TC001209	Fructose-1%2C6-bisphosphatase 1-like Protein	FBgn0032820	Fructose bisphosphatase	fructose metabolism	11
LG2	12710438	TC000350	-	FBgn0039638	dim γ-tubulin 6	mitosis	12
LG2	12710438	TC001210	Coatomer subunit epsilon-like Protein	FBgn0027496	Coat Protein (coatomer) ε	Golgi vesicle-mediated transport	11
LG3	25378890	TC034629	Hepatic leukemia factor-like protein	FBgn0016694	PAR-domain protein 1	lipid metabolism; circadian clock	13
LG4	9240313	TC007111	-	FBgn0052685	ZAP3	telomere maintenance	11
LG4	9240313	TC008371	Glutaredoxin	FBgn0030584	Glutaredoxin 5	iron-sulphur cluster assembly	14
LG4	9240313	TC008372	Leucine--tRNA ligase%2C cytoplasmic-like Protein	FBgn0284253	Leucyl-tRNA synthetase	would healing	15
LG6	9671781	TC014856	Nucleoredoxin-like Protein	-	-	-	-
LG7	1184873	TC009442		FBgn0034563	CG15649	-	-
LG7	1184873	TC009377		FBgn0083951	CG34115	-	-
LG7	1184873	TC009441	Collagen type IV alpha-3-binding protein-like Protein	FBgn0027569	ceramide transfer protein	ageing; oxidative stress	16
LG7	1184873	TC009376	Protein RIC1 homolog-like Protein	FBgn0028500	RIC1 homolog	eye development; synapse functioning	17
LG8	12459002	TC006646	-	FBgn0038819	Cuticular protein 92F	cuticle development	18
LG8	12459002	TC034151	-	-	-	-	-

LG8	12459002	TC005479	Polypeptide N-acetylgalactosaminyltransferase	FBgn0050463	Polypeptide N-Acetylgalactosaminyltransferase 9	sexual reproduction, acessory gland protein	19
LG8	12459002	TC034154	-	-	-	-	-
LG8	12459002	TC006650	-	-	-	-	-
LG9	8113123	TC034351	-	FBgn0261836	Muscle-specific protein 300 kDa	oogenesis; flight; locomotion	20
LG9	8843888	TC034357	-	FBgn0287478	Calmodulin-binding transcription activator	memory formation; courtship behaviour	21
LGX	3236618	TC013602	Vitellogenin-like Protein	-	-	-	-
LGX	3236618	TC013601	Putative DNA mismatch repair protein Msh6-like Protein	FBgn0036486	Msh6	meiotic DNA mismatch repair	22
LGX	3236618	TC014211	Neuropeptide Y receptor-like Protein	FBgn0038880	SIFamide receptor	sex discrimination, mating behaviour, neuropeptide signalling	23
LGX	3470189	TC014225	Transcription factor E3-like Protein	FBgn0263112	Mitf	starvation response; llipid catabolism	24
LGX	3470189	TC014226	Kinesin-like protein KIF13A	FBgn0019968	Kinesin heavy chain 73	synaptic function; olfactory learning	25

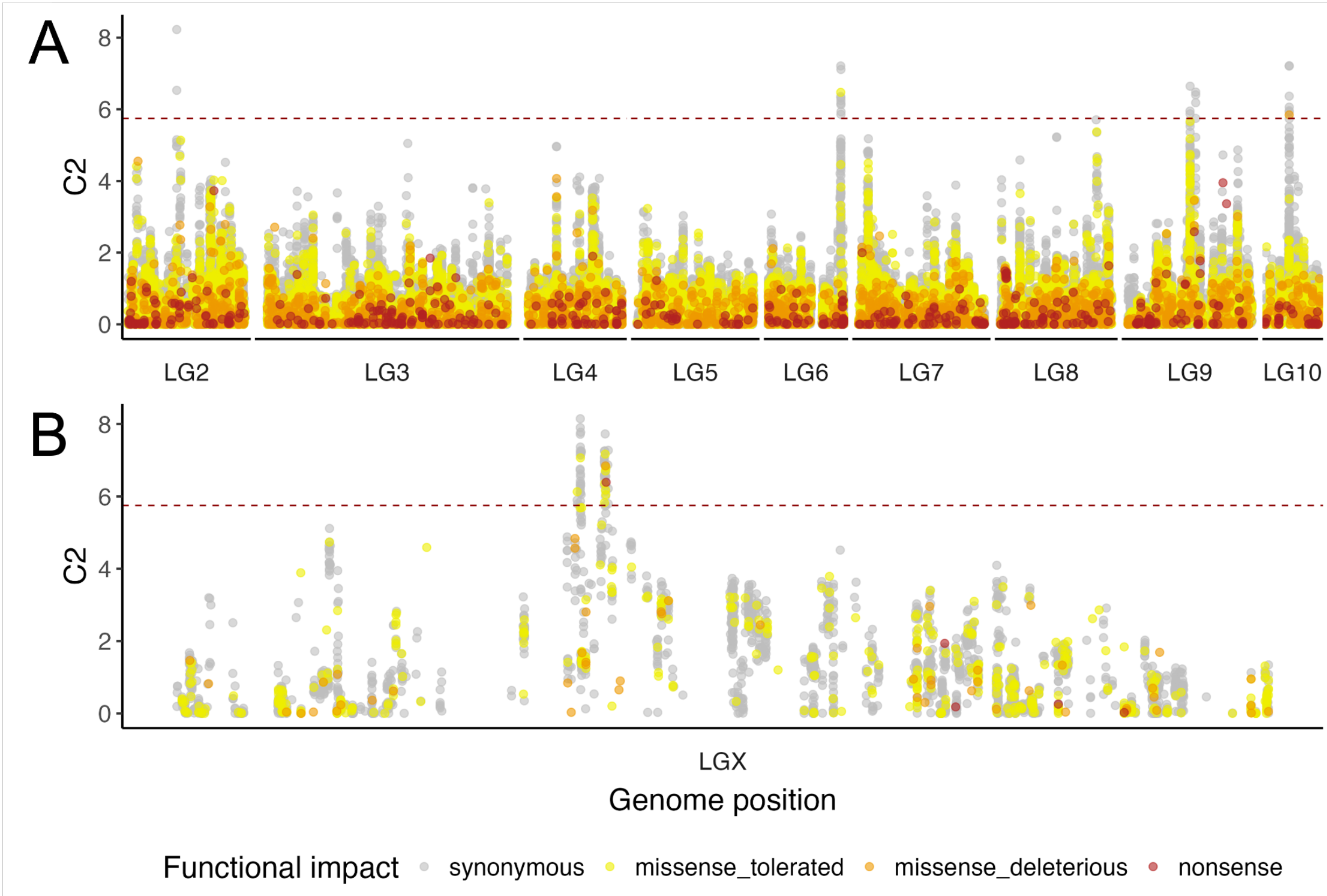

Figure S6. Genomic loci showing allele frequency differentiation associated with sexual selection regime in experimentally evolving Tribolium castaneum populations, coloured by functional impact. Values of the C2 statistic generated by BayPass analysis (26) measuring SNP-wise associations with the two sexual selection regimes (polyandrous vs. monandrous) while accounting for shared population history. The dotted line shows the 0.999 quantile of C2 values from a BayPass run on a pseudo-observed dataset of putatively neutral SNPs. Colours denote the functional impact derived via protein logic (SnpEff; 27) and conservance score for non-synonymous variants (SIFT; 28). Separate analyses were performed for **A)** autosomal linkage groups, and **B)** the X chromosome (LGX).

References

1. Y. Xue, J. Prado-Martinez, P. H. Sudmant, V. Narasimhan, et al., Mountain gorilla genomes reveal the impact of long-term population decline and inbreeding. Science 348, 242–245 (2015)

2. N. Dussex, H. E. Morales, C. Grossen, L. Dalén, C. van Oosterhout, Purging and accumulation of genetic load in conservation. Trends Ecol. Evol. 38, 961–969 (2023)

3. A. J. Lumley, Ł. Michalczyk, J. J. N. Kitson, L. G. Spurgin, et al., Sexual selection protects against extinction. Nature 522, 470–473 (2015)

4. S. J. Marygold, J. Roote, G. Reuter, A. Lambertsson, et al., The ribosomal protein genes and Minute loci of Drosophila melanogaster. Genome Biol. 8, R216 (2007)

5. F. Chen, V. Archambault, A. Kar, P. Lio, et al., Multiple protein phosphatases are required for mitosis in *Drosophila*. Curr. Biol. 17, 293–303 (2007)

6. J. Yu, Q. Zheng, Z. Li, Y. Wu, et al., CG6015 controls spermatogonia transit-amplifying divisions by epidermal growth factor receptor signaling in *Drosophila* testes. Cell Death Dis. 12, 491 (2021)

7. T. Nagao, K. Endo, H. Kawauchi, U. Walldorf, K. Furukubo-Tokunaga, Patterning defects in the primary axonal scaffolds caused by the mutations of the extradenticle and homothorax genes in the embryonic *Drosophila* brain. Dev. Genes Evol. 210, 289–299 (2000)

8. M. Kurihara, K. Komatsu, K. Awane, Y. H. Inoue, Loss of Histone Locus Bodies in the Mature Hemocytes of Larval Lymph Gland Result in Hyperplasia of the Tissue in *mxc* Mutants of *Drosophila*. Int. J. Mol. Sci. 21, E1586 (2020)

9. Q. Jin, Y. Wang, H. Yin, H. Jiang, Two clip-domain serine protease homologs, cSPH35 and cSPH242, act as a cofactor for prophenoloxidase-1 activation in Drosophila melanogaster. Front. Immunol. 14, 1244792 (2023).

10. C. Mendoza-Topaz, F. Urra, R. Barría, V. Albornoz, D. Ugalde, U. Thomas, E. D. Gundelfinger, R. Delgado, M. Kukuljan, P. D. Sanxaridis, S. Tsunoda, M. F. Ceriani, V. Budnik, J. Sierralta, DLGS97/SAP97 is developmentally upregulated and is required for complex adult behaviors and synapse morphology and function. J. Neurosci. 28, 304–314 (2008).

11. P. Gaudet, R. Chisholm, T. Berardini, E. Dimmer, S. R. Engel, P. Fey, et al., The Gene Ontology's Reference Genome Project: A unified framework for functional annotation across species. PLoS Comput. Biol. 5, e1000431 (2009).

12. G. A. Pavlova, A. V. Razuvaeva, J. V. Popova, E. N. Andreyeva, L. A. Yarinich, M. O. Lebedev, C. Pellacani, S. Bonaccorsi, M. P. Somma, M. Gatti, A. V. Pindyurin, The role of Patronin in Drosophila mitosis. BMC Mol. Cell Biol. 20, 7 (2019).

13. S. A. Cyran, A. M. Buchsbaum, K. L. Reddy, M. C. Lin, N. R. J. Glossop, P. E. Hardin, M. W. Young, R. V. Storti, J. Blau, vrille, Pdp1, and dClock form a second feedback loop in the Drosophila circadian clock. Cell 112, 329–341 (2003).

14. Z. Marelja, S. Leimkühler, F. Missirlis, Iron sulfur and molybdenum cofactor enzymes regulate the Drosophila life cycle by controlling cell metabolism. Front. Physiol. 9, 50 (2018).

15. I. Campos, J. A. Geiger, A. C. Santos, V. Carlos, A. Jacinto, Genetic screen in Drosophila melanogaster uncovers a novel set of genes required for embryonic epithelial repair. Genetics 184, 129–140 (2010).

16. R. P. Rao, C. Yuan, J. C. Allegood, S. S. Rawat, M. B. Edwards, X. Wang, A. H. Merrill, U. Acharya, J. K. Acharya, Ceramide transfer protein function is essential for normal oxidative stress response and lifespan. Proc. Natl. Acad. Sci. U.S.A. 104, 11364–11369 (2007).

17. C. Tong, T. Ohyama, A. C. Tien, A. Rajan, C. M. Haueter, H. J. Bellen, Rich regulates target specificity of photoreceptor cells and N-cadherin trafficking in the Drosophila visual system via Rab6. Neuron 71, 447–459 (2011).

18. M. V. Karouzou, Y. Spyropoulos, V. A. Iconomidou, R. S. Cornman, S. J. Hamodrakas, J. H. Willis, Drosophila cuticular proteins with the R&R consensus: annotation and classification with a new tool for discriminating RR-1 and RR-2 sequences. Insect Biochem. Mol. Biol. 37, 754–760 (2007).

19. K. Ravi Ram, M. F. Wolfner, Seminal influences: Drosophila Acps and the molecular interplay between males and females during reproduction. Integr. Comp. Biol. 47, 427–445 (2007).

20. J. Yu, D. A. Starr, X. Wu, S. M. Parkhurst, Y. Zhuang, T. Xu, R. Xu, M. Han, The KASH domain protein MSP-300 plays an essential role in nuclear anchoring during Drosophila oogenesis. Dev. Biol. 289, 336–345 (2006).

21. K. Sato, M. T. Ahsan, M. Ote, M. Koganezawa, D. Yamamoto, Calmodulin-binding transcription factor shapes the male courtship song in Drosophila. PLoS Genet. 15, e1008309 (2019).

22. S. J. Radford, M. M. Sabourin, S. McMahan, J. Sekelsky, Meiotic recombination in Drosophila Msh6 mutants yields discontinuous gene conversion tracts. Genetics 176, 53–62 (2007).

23. A. Sellami, J. A. Veenstra, SIFamide acts on fruitless neurons to modulate sexual behavior in Drosophila melanogaster. Peptides 74, 50–56 (2015).

24. V. Bouché, A. P. Espinosa, L. Leone, M. Sardiello, A. Ballabio, J. Botas, Drosophila Mitf regulates the V-ATPase and the lysosomal-autophagic pathway. Autophagy 12, 484–498 (2016).

25. K. Tsurudome, K. Tsang, E. H. Liao, R. Ball, J. Penney, J. S. Yang, F. Elazzouzi, T. He, A. Chishti, G. Lnenicka, E. C. Lai, A. P. Haghighi, The Drosophila miR-310 cluster negatively regulates synaptic strength at the neuromuscular junction. Neuron 68, 879–893 (2010).

26. L. Olazcuaga, A. Loiseau, H. Parrinello, M. Paris, et al., A Whole-Genome Scan for Association with Invasion Success in the Fruit Fly *Drosophila suzukii* Using Contrasts of Allele Frequencies Corrected for Population Structure. Mol. Biol. Evol. 37, 2369–2385 (2020).

27. P. Cingolani, A. Platts, L. L. Wang, M. Coon, et al., A program for annotating and predicting the effects of single nucleotide polymorphisms, SnpEff: SNPs in the genome of *Drosophila melanogaster* strain w1118; iso-2; iso-3. Fly (Austin) 6, 80–92 (2012).

28. R. Vaser, S. Adusumalli, S. N. Leng, M. Sikic, P. C. Ng, SIFT missense predictions for genomes. Nat. Protoc. 11, 1–9 (2016).
